# Mechanisms of CP190 Interaction with Architectural Proteins in Drosophila Melanogaster

**DOI:** 10.3390/ijms222212400

**Published:** 2021-11-17

**Authors:** Marat Sabirov, Anastasia Popovich, Konstantin Boyko, Alena Nikolaeva, Olga Kyrchanova, Oksana Maksimenko, Vladimir Popov, Pavel Georgiev, Artem Bonchuk

**Affiliations:** 1 Department of the Control of Genetic Processes, Institute of Gene Biology Russian Academy of Sciences, 34/5 Vavilov St., 119334 Moscow, Russia; greencapers@yandex.ru (M.S.); anast.popovich@gmail.com (A.P.); olgina73@gmail.com (K.B.); maksog@mail.ru (O.K.); 2 Center for Precision Genome Editing and Genetic Technologies for Biomedicine, Institute of Gene Biology, Russian Academy of Sciences, 34/5 Vavilov St., 119334 Moscow, Russia; boiko_konstantin@inbi.ras.ru; 3 Research Center of Biotechnology, Russian Academy of Sciences, Leninsky Prospekt, 33, Bld. 2, 119071 Moscow, Russia; vpopov@inbi.ras.ru; 4 National Research Center “Kurchatov Institute”, 123182 Moscow, Russia; aishome@mail.ru

**Keywords:** Pita, Su(Hw), CTCF, chromatin insulator, transcription, BTB domain, C2H2 proteins

## Abstract

Most of the known Drosophila architectural proteins interact with an important cofactor, CP190, that contains three domains (BTB, M, and D) that are involved in protein–protein interactions. The highly conserved N-terminal CP190 BTB domain forms a stable homodimer that interacts with unstructured regions in the three best-characterized architectural proteins: dCTCF, Su(Hw), and Pita. Here, we identified two new CP190 partners, CG4730 and CG31365, that interact with the BTB domain. The CP190 BTB resembles the previously characterized human BCL6 BTB domain, which uses its hydrophobic groove to specifically associate with unstructured regions of several transcriptional repressors. Using GST pull-down and yeast two-hybrid assays, we demonstrated that mutations in the hydrophobic groove strongly affect the affinity of CP190 BTB for the architectural proteins. In the yeast two-hybrid assay, we found that architectural proteins use various mechanisms to improve the efficiency of interaction with CP190. Pita and Su(Hw) have two unstructured regions that appear to simultaneously interact with hydrophobic grooves in the BTB dimer. In dCTCF and CG31365, two adjacent regions interact simultaneously with the hydrophobic groove of the BTB and the M domain of CP190. Finally, CG4730 interacts with the BTB, M, and D domains of CP190 simultaneously. These results suggest that architectural proteins use different mechanisms to increase the efficiency of interaction with CP190.

## 1. Introduction

A large family of transcription factors found in most higher eukaryotes comprises proteins containing a common highly conserved protein–protein interaction domain known as the Broad-complex, Tramtrack, and Bric-à-brac/poxvirus (BTB) domain [1,2]. Transcription factors with the BTB domain (BTB TFs) have diverse functions in transcriptional regulation, chromatin remodeling, and protein degradation [3]. Studies in mammalian systems have shown that BTB TFs are implicated in cancer and musculoskeletal diseases [4]. A considerable fraction of the BTB TFs, which are well characterized in *Drosophila melanogaster*, mice, and humans, also contain C2H2-type zinc-finger domains (C2H2) [5]. The clusters of C2H2 domains in TFs are usually involved in high-affinity binding to specific sites on chromatin [6,7]. 

The human BCL6 protein contains the best-characterized BTB domain, which exists as a stable obligate homodimer [8]. The dimer interface forms two extended grooves that serve as docking sites for three corepressors: SMRT, NCOR, and BCOR [8,9,10]. SMRT and NCOR bind to the BCL6 BTB groove with similar peptide sequences, while BCOR binds to the same grooves of the BTB dimers of BCL6 using a completely different peptide [8,9]. In mammals, several BTB proteins function as Cullin 3 adaptors that are involved in ubiquitination of targeted proteins [11,12,13,14,15]. The structural basis for this interaction was shown for several proteins, including the BTB-Kelch and BTB-MATH proteins, which contain an adjacent BACK domain [16,17,18,19]. Taken together, the available data show that BTB domains are involved in recruiting different complexes to chromatin.

In Drosophila, several BTB-C2H2 transcription factors that are involved in transcription regulation and chromatin remodeling have been characterized [3,20]. The structure was resolved for the BTB domain of the protein CP190 [21,22], which binds predominantly to promoters of housekeeping genes and insulators [23,24,25]. CP190 (a 1096-amino acid protein) contains an N-terminal BTB/POZ domain, an aspartic acid-rich D-region, four C2H2 zinc finger motifs, and a C-terminal E-rich domain [26,27]. In addition to these motifs, CP190 also contains a centrosomal-targeting domain (M) responsible for its localization to centrosomes during mitosis [26]. Like those of the BCL6 and PLZF proteins, the N-terminal BTB/POZ domain of CP190 forms stable homodimers [21,27,28]. A unifying chromatin feature for most of the CP190-bound regions is an increase in histone acetylation, suggesting the role of this factor in chromatin opening and transcription activation [24,29,30,31,32]. It was shown that CP190 is involved in the recruitment of the NURF, dREAM, and SAGA complexes to the chromatin [23,30,32,33]. Several lines of evidence also suggest a possible role of CP190 in the organization of chromosome architecture by bridging together distantly located sites [21,33,34]. 

Four C2H2 domains located in the center of CP190 seem to be involved in protein–protein interactions rather than in DNA binding [27]. Most evidently, CP190 is recruited to chromatin via interaction with DNA-binding transcription factors [20]. 

In particular, CP190 interacts with a large group of architectural proteins that have clusters of zinc-finger domains of C2H2 type [35,36,37,38]. A characteristic feature of architectural proteins is the ability to specifically bind to long (12–15 bp) DNA motifs via four or five C2H2 domains located in clusters [39]. Usually, Drosophila architectural proteins contain the N-terminal domains that predominantly form homodimers [7,39]. These domains are essential for specific distance interactions between the chromatin sites bound by architectural proteins [40]. In addition, there is some evidence that CP190 is also involved in protein–protein interactions that establish chromatin architecture and support enhancer–promoter communication [21,33,41,42,43]. 

The BTB domain of CP190 is required for interaction with the architectural proteins Su(Hw) [44], Pita [35,45], and dCTCF [46]. With another group of architectural proteins that includes ZIPIC and Opbp, the CP190 protein interacts via its centrosomal-targeting domain (M) [35,36]. The architectural proteins Su(Hw), Pita, and dCTCF in cooperation form boundaries/insulators in the Bithorax complex [47]. The CP190 protein is important for blocking cross-interaction between adjacent regulatory domains [45]. 

The goal of this work was to identify novel potential architectural proteins with clusters of C2H2 domains that interact with the BTB domain of CP190 and to understand the mechanisms of CP190 recruitment on the chromatin sites by the C2H2 proteins. By screening a library of the Drosophila C2H2 proteins in a yeast two-hybrid (Y2H) assay against the BTB domain of CP190, we identified two new proteins interacting with this domain. A crystal structure of the CP190 BTB showed the existence of a hydrophobic groove like that observed in the BCL6 BTB. Point mutations in the hydrophobic groove affected interactions of the CP190 BTB with the C2H2 proteins. By using GST pull-down and Y2H assays, we found that the C2H2 proteins use different strategies to increase the affinity of interaction with CP190.

## 2. Results

### 2.1. Identification of Two New C2H2 Proteins That Interact with the BTB Domain of CP190

To identify new potential architectural proteins that interact with the BTB domain of CP190, we used a library of 154 Drosophila C2H2 proteins attached to the activation domain of GAL4 (Supplementary Materials Table S1). These proteins were selected based on the presence of a cluster of at least five C2H2 domains that can participate in the specific recognition of long DNA motifs.

**Figure 1:**
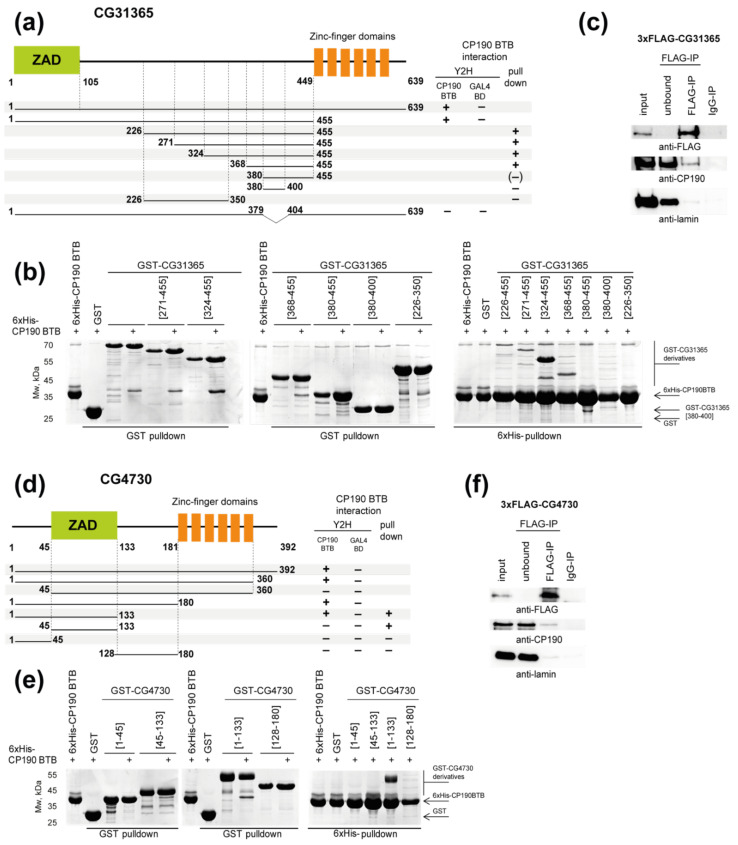
Two new C2H2 proteins that interact with the BTB domain of CP190. (**a**) Summary of the mapping of the interaction between CG31365 and CP190 proteins in pull-down and yeast two-hybrid (Y2H) assays. An uncertain interaction is shown in brackets. For Y2H, different fragments of CG31365 were fused with the GAL4 activation domain and tested for interaction with the CP190 BTB domain fused to the GAL4 DNA-binding domain (GAL4 BD). GAL4 BD serves as the negative control. The growth assay yeast plates are shown in Supplementary Materials Figure S2. (**b**) Mapping of the interaction between CG31365 and CP190 proteins using pull-down assays. The uncropped images are shown in the Supplementary Materials Figure S3. (**c**) Co-immunoprecipitation of CP190 and CG31365 tagged with FLAG in extracts from Drosophila S2 cells. Total extracts were immunoprecipitated with FLAG antibodies. The immunoprecipitates (IPs) were analyzed by Western blotting. The uncropped images are shown in Supplementary Materials Figure S1. (**d**) Summary of the mapping of the interaction between CG4730 and CP190 proteins in pull-down and Y2H assays. Designations are the same as in panel (**a**). (**e**) Mapping of the interaction between CG4730 and CP190 proteins using pull-down assays. The uncropped images are shown in the Supplementary Materials Figure S3. (**f**) Co-immunoprecipitation of CP190 and CG4730 tagged with FLAG in extracts from Drosophila S2 cells. Total extracts were immunoprecipitated with FLAG antibodies. The immunoprecipitates (IPs) were analyzed by Western blotting. The uncropped images are shown in the Supplementary Materials Figure S1.

Library screening using a Y2H assay identified all known to date CP190 BTB-interacting proteins (Pita, dCTCF, and Su(Hw)) and two novel proteins: CG4730 and CG31365. Both proteins contain an N-terminal Zinc finger-Associated Domain (ZAD) separated with a spacer from a tandem array of zinc-finger domains (Figure 1a,d). Interaction of these proteins with CP190 was confirmed by co-immunoprecipitation of CP190 and FLAG-tagged CG4730 or CG31365 transfected in Drosophila S2 cells (Figure 1c,f, Supplementary Materials Figure S1). 

To map the regions of CG4730 and CG31365 involved in the interaction with the CP190 BTB domain, we carried out Y2H and pull-down assays (Figure 1a,b, Supplementary Materials Figure S2). The CG31365 protein (639 aa) consists of the ZAD domain (13–87 aa), a spacer (88–448 aa, predicted to be mostly unstructured), and an array of six C2H2 domains (449–619 aa) (Figure 1a). Using a Y2H assay, the CP190-interacting region was mapped to the 1–455 aa region of CG31365. Using a pull-down assay with bacterially expressed protein domains, the BTB-interacting domain was mapped between aa 368 and 455 in the unstructured region (Figure 1a,b). The 380–400 aa region does not bind CP190 in vitro, suggesting that it lacks some residues important for the interaction. At the same time, deletion of residues 379–404 from the full-length protein completely impaired the interaction with the CP190 BTB domain in the Y2H assay (Figure 1a), indicating that it is essential but not sufficient for binding. 

The CG4730 protein has short N-terminal and C-terminal unstructured regions (1–45 aa and 347–392 aa), a ZAD (46–125 aa), and an array of six C2H2 domains (181–346 aa) separated by a 54 aa spacer (Figure 1d). In a Y2H system, the BTB domain was found to interact with the 1–45 aa region preceding the ZAD (Figure 1d). The ZAD domain is also essential for this interaction but is not sufficient by itself. The same results were observed in a pull-down assay except that the ZAD domain also displayed weak binding to the BTB domain (Figure 1d,e). It seems likely that the interaction of CG4730 with CP190 BTB is relatively weak and that the dimerization ability of the ZAD is critical to improve the affinity of the interaction.

In general, the CP190 BTB-binding motifs in dCTCF, Pita, Su(Hw), and CG31365 are conserved in Drosophila species but not in other insects, including Diptera (Supplementary Materials Figures S4 and S5a). In the case of CG4730, the BTB-binding region is not conserved in orthologs from *D. virilis*, *D. ananassae*, and *D. mojavensis* (Supplementary Materials Figures S4 and S5b). Since CG4730 orthologs were not found in some Drosophila species (*D. willistoni, D. grimshawi* (Supplementary Materials Figure S5b)), this protein is likely the result of a recent gene duplication event. The N-terminal region that interacts with CP190 BTB is present only in the CG4730 orthologs from species that are closely related to *D. melanogaster*. It is more likely that it is a newly arising interaction.

### 2.2. The CG31365 and CG4730 Proteins Interact Not Only with the BTB, but Also with the M and D Domains of CP190

**Figure 2:**
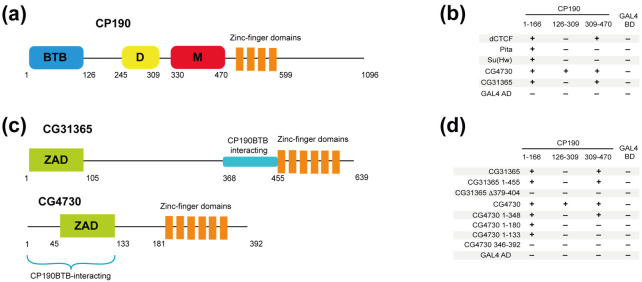
CP190 uses different domains to interact with C2H2 proteins. (**a**) Schematic presentation of the CP190 domain structure. (**b**) Determination of CP190 domains involved in the interaction with C2H2 proteins. (**c**) Schematic presentation of CG31365 and CG4730 domain structures with CP190 BTB domain-interacting regions depicted. (**d**) Mapping of regions in CG4730 and CG31365 that interact with CP190 in a yeast two-hybrid assay. Full-length and various fragments of CG4730 and CG31365 were fused with the GAL4-activating domain (GAL4 AD) and tested for interaction with the CP190 domains fused to the GAL4 DNA-binding domain (GAL4 BD). The growth assay yeast plates are shown in the Supplementary Materials Figure S7.

In addition to the BTB domain, the D and M domains in CP190 (Figure 2a) were also shown to interact with the architectural proteins. The dCTCF protein interacts simultaneously with the BTB and M domains of CP190 [48], while the ZIPIC protein interacts only with the M domain [35]. The Opbp protein interacts with the aspartic acid-rich D-region (D domain, 245–309 aa) of CP190 [36]. Interestingly, both CP190 regions are responsible for CP190 localization to centrosomes during mitosis [22,26]. Moreover, both domains contain conserved amino acid stretches (Supplementary Materials Figure S6).

We asked whether CP190-interacting C2H2 proteins are able to interact with the D and M domains in a Y2H assay (Figure 2b). It was confirmed that dCTCF interacts with the CP190 309–470 sequence (M domain). In contrast, the Su(Hw) and Pita proteins do not interact with the D and M domains of CP190 in the Y2H assay. Like dCTCF, CG31365 was shown to interact only with the M domain (Figure 2b), whereas CG4730 interacts with both the D and the M domains.

Next, we mapped the D- and M-interacting regions in the CG31365 and CG4730 proteins (Figure 2c). In CG31365, 1–455 aa, which contain the ZAD and the spacer region, were sufficient for the interaction with the M domain. Interestingly, the deletion of residues 379–404, which abolishes the interaction with the BTB domain (Figure 2c), also impairs the interaction with the CP190 M domain (Figure 2d).

In the case of CG4730, only full-length protein was able to interact with the M and D domains, suggesting a possible cooperation between several domains in this interaction (Figure 2d). Thus, the CG4730 and CG31365 proteins interact not only with the BTB domain, but also with additional domains of CP190. 

### 2.3. Identification of Key Amino Acids in BTB Involved in Interactions with C2H2 Proteins

**Figure 3:**
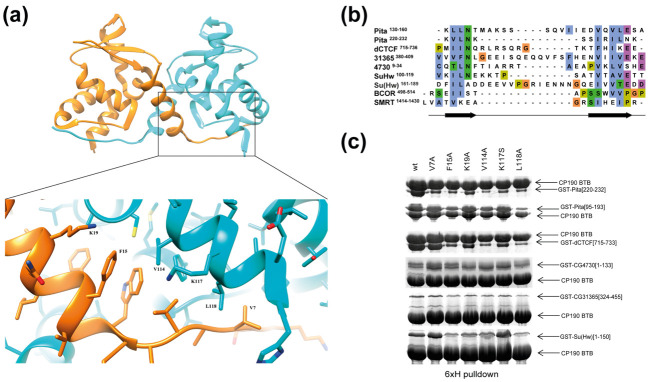
The hydrophobic groove of the CP190 BTB domain is involved in interaction with most of the C2H2 proteins. (**a**) 1.4 Å crystal structure of the CP190 BTB domain dimer with an enlarged view of the peptide-binding groove. Residues subjected to mutagenesis are designated. (**b**) Alignment of core sequences in the C2H2 proteins that interact with the CP190 BTB domain. Amino acids are colored according to their properties. The positions of the predicted beta strands are shown. (**c**) Testing of the impact of point mutations within the CP190 BTB peptide-binding groove on the interaction with C2H2 proteins using a pull-down assay. The uncropped images are shown in the Supplementary Materials Figure S9.

Next, we asked how the CP190 BTB domain interacts with the C2H2 proteins. For this, we examined the crystal structure of the CP190 BTB domain at 1.4 Å resolution (Figure 3a). In accordance with previous reports [21,22], we found that the BTB domain of CP190 exists in a homodimeric state in a crystal and has a typical overall architecture composed of a cluster of six alpha helices capped on the C-terminal end by three beta sheets as well as one beta strand on the N-terminus. Although solved with better resolution compared to the other known structures of the CP190 BTB domain (PDB codes 4U77 and 5EUP), this new structure demonstrates only minor differences (see Supplementary Materials Data for a detailed description). 

**Figure 4:**
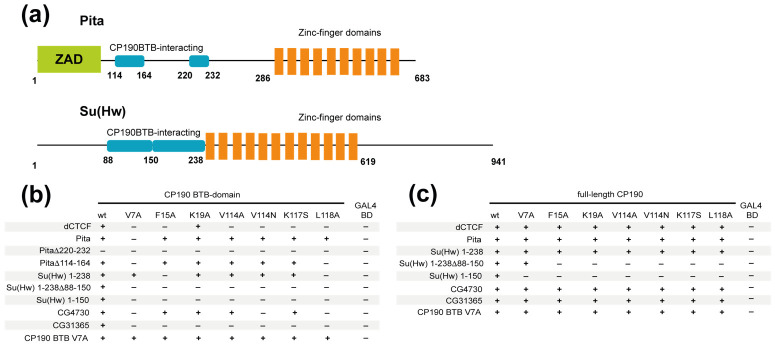
Testing of interactions between the C2H2 proteins and the mutant variants of the CP190 protein in a yeast two-hybrid assay. (**a**) Schematic representation of Pita and Su(Hw) domain structures with CP190 BTB domain-interacting regions depicted. (**b**) Interaction of the C2H2 proteins with CP190 BTB domains bearing single amino acid substitutions within the peptide-binding groove. (**c**) Interaction of the C2H2 proteins with full-length CP190 proteins bearing single amino acid substitutions within the BTB domain. CP190 BTB domain and full-length proteins were fused to the GAL4 DNA-binding domain (GAL4 BD); C2H2 proteins were fused with the GAL4 activation domain. The growth assay yeast plates are shown in the Supplementary Materials Figures S10 and S11.

Previously, we mapped the 13-aa region (220–232 aa) in Pita and the 19-aa region (715–733 aa) in dCTCF that are critical for interaction with the BTB domain [45,48]. Pita also has a second minor region (114–164 aa) that interacts with the BTB domain with low affinity [45] (Figure 4a). Interestingly, Su(Hw) also has two adjacent regions, mapped to aa 88–150 and 150–238, that bind to the BTB domain [44] (Figure 4a). In Su(Hw), the aa 150–238 region can be further shortened to aa 150–187 by excluding the adjacent acidic region after residue 187, which separates the conserved sequence from the first C2H2 zinc-finger domain (Supplementary Materials Figure S4). Unfortunately, crystallization trials with small high-affinity BTB-binding peptides derived from Pita and dCTCF (Supplementary Materials Table S2) were unsuccessful: in all cases, we obtained BTB crystals lacking the peptide.

The structure of the CP190 BTB domain is similar to that of the well-characterized BTB domain of the human BCL6 protein [8]. Crystal structures of the BCL6 BTB with peptides of the BCOR and SMRT proteins were resolved [8,9,10]. These structures showed that the BCOR and SMRT peptides interact with a hydrophobic groove of the BTB domain. The CP190 BTB domain has a similar hydrophobic groove formed by β1-α1 and by α6 of the adjacent subunit (Figure 3a). Notably, BCOR and SMRT BTB-binding peptides lack similarity in their primary amino acid sequences and no binding motif can be described, since most of the conserved contacts are main-chain interactions, which are primarily polar [9]. Both peptides tend to form beta strands when bound to the BCL6 BTB domain. A comprehensive examination of the CP190 BTB-binding regions revealed the presence of two potential beta strands separated by polar sequences of 5–15 aa in length (Figure 3b). For instance, the high-affinity peptide from the Pita protein (220–232 aa) contains two almost identical repeats: KVLNK and RILNK. Pita^114–164^ contains similar but imperfect copies of the predicted beta strands (KLLNT and QVLES). Similar motifs can be found in the core BTB-binding sequences of other CP190 BTB-interacting proteins (Figure 3b). 

By using the semi-flexible molecular docking approach Galaxy PepDock [49], we found that the hydrophobic groove of the CP190 BTB might interact with unstructured peptides (Supplementary Materials Figure S8a). The surface of the BTB domain lacks other large hydrophobic interfaces (Supplementary Materials Figure S8b); therefore, the groove is the most likely candidate for interaction with unstructured peptides. To test this model, a set of point mutations along the peptide-binding groove of the CP190 BTB domain was designed: alanine substitutions of hydrophobic residues (V7A, F15A, V114A, L118A), a mutation that would create a steric clash (V114N), and substitutions of charged amino acids (K19A, K117S) (Figure 3a). 

Using glutaraldehyde crosslinking of thioredoxin-fused BTB domains bearing amino acid substitutions, we confirmed that, like the wild-type (*wt*) BTB domain, all mutant BTBs form dimers (Supplementary Materials Figure S8c) and have good solubility after bacterial expression (Supplementary Materials Figure S8d). Next, the mutant variants of the BTB domain were tested for the interaction with the previously mapped peptides in a pull-down assay (Figure 3c). In this assay, the interaction of the Pita^220–232^, dCTCF^715–733^, and Su(Hw)^1–150^ peptides with the mutant BTB domains was reduced in most cases, confirming that these peptides interact with large hydrophobic interfaces in the groove. The second BTB-binding region of Su(Hw)^150–187^ apparently has weak affinity to the CP190 BTB in the pull-down assay (Supplementary Materials Figure S7e) and was thus tested for interaction with mutant BTB domains using only the Y2H assay (Figure 4a). Notably, none of these peptides can effectively interact with BTB^L118A^. In contrast, BTB^V7A^ effectively interacts with most of the tested peptides, with the exception of Pita^220–232^. All mutations in the CP190 BTB-binding groove reduced their affinity for the Pita^220–232^ peptide. In contrast, the Pita^95–193^ containing the second BTB-binding region was sensitive only to L118A substitution. Thus, the GST pull-down (Figure 3c) experiments support a key role for the hydrophobic groove in the interaction of the CP190 BTB with Su(Hw), dCTCF, and Pita.

In the pull-down assays, the interactions of the CP190 BTB with CG4730^1–133^ and CG31365^324–455^ were almost insensitive to substitutions within the peptide-binding groove. These results suggest that either these peptides interact with CP190 BTB by a different mechanism, or the pull-down assay is not sensitive in these cases.

### 2.4. The C2H2 Proteins Use Different Approaches to Increase the Affinity of Interaction with CP190

To further study how C2H2 proteins interact with the CP190 BTB domain, we tested the interaction with wild-type and mutant variants of BTB in the Y2H assay (Figure 4b). We confirmed that the BTB domain effectively interacts with the Su(Hw) N-terminal domain and the Pita, dCTCF, CG31365, and CG4730 proteins. Unexpectedly, we found that all mutant variants of the BTB domain retain the ability to interact with the Pita protein, in contrast to the results obtained in the pull-down assay (Figure 3c). However, Pita^∆220–232^ cannot interact with even the wild-type BTB domain, demonstrating that the 114–164 aa region has only weak affinity for the BTB domain. Pita^∆114–164^ was also able to interact with all mutant CP190 BTB variants except V7A and L118A, indicating that the 114–164 aa region plays only an auxiliary role in the interaction between CP190 and Pita. In a Y2H assay, dCTCF and CG31365 interacted only with the wild-type CP190 BTB but not with most of the BTB mutants (dCTCF is able to interact only with K19A mutant BTB), demonstrating the important role of all amino acids in the hydrophobic groove in these interactions. The tested proteins (especially CG31365) showed a higher sensitivity to CP190 BTB mutations in the Y2H assay than in the pull-down assay. This difference can be explained by the significantly lower concentrations of the tested proteins in yeast cells compared to bacteria.

The N-terminal domain of Su(Hw), which contains both CP190 BTB-interacting sequences, is able to interact with wild-type as well as with all mutant derivatives of CP190 in the Y2H assay (Figure 4b). However, each of the two Su(Hw) CP190-binding regions can separately interact only with the wild-type BTB domain. Thus, the two domains in Su(Hw) have additive roles in the formation of high-affinity interactions with the BTB domain. The CG4730 is able to interact with all variants of the CP190 BTB. However, we cannot distinguish between the roles of the N-terminal peptide and the adjacent ZAD that form homodimers. 

Next, we tested the interactions between the C2H2 proteins and full-length CP190 proteins carrying different substitutions in the BTB domain (Figure 4c). As expected, all proteins were able to interact with all mutant variants of CP190. These results suggest that the adjacent regions (BTB, D, and M) additively contribute to the high-affinity association between CP190 and the C2H2 proteins. 

## 3. Discussion

In this study, we identified two novel C2H2 proteins that interact with the BTB domain of CP190. Both proteins belong to the large group of transcription factors (ZAD-C2H2) that contain ZADs at the N-termini and arrays of C2H2 domains at the C-termini. The ZAD is usually located at the very N-terminus of a C2H2 protein and forms homodimers. At least four ZAD-C2H2 proteins (Pita, Zw5, ZAF1, and ZIPIC) have been assigned to the class of architectural proteins because they can support specific distance interactions between regulatory elements and function as part of insulators [35,40,50,51,52]. The ZAD-C2H2 protein, named M1BP, in cooperation with CP190 participate in the formation of TAD boundaries [53,54]. Binding sites for ZAD-C2H2 proteins are predominantly located in the region of active promoters [40,51,53,55]. It is assumed that they participate in the formation of open chromatin in the region of promoters and contribute to the recruitment of transcription factors that are not capable of binding with high specificity to DNA [39,56].

Our mutagenic analysis of the CP190 BTB domain suggests that the hydrophobic groove is the main surface for interaction with the Pita, dCTCF, Su(Hw), and CG31365 proteins. Previous structural analysis of complexes between peptides and the BCL6 BTB showed that very different peptide sequences can be involved in the interaction with the hydrophobic groove. Structure-based alignment suggests that many polar contacts are conserved; however, they are mostly main-chain interactions, so they use various residues of BCL6-binding peptides [9]. Non-conserved contacts, in contrast, are mainly apolar and form side-chain interactions that are involved in further stabilization of the interfaces. It is highly likely that a similar mechanism is exploited in the CP190 BTB domain interaction with C2H2 proteins, as within conserved sequence stretches, we were able to find double hydrophobic patches with a predicted propensity to form beta strands flanked by polar residues; however, no actual sequence motif can be described.

**Figure 5:**
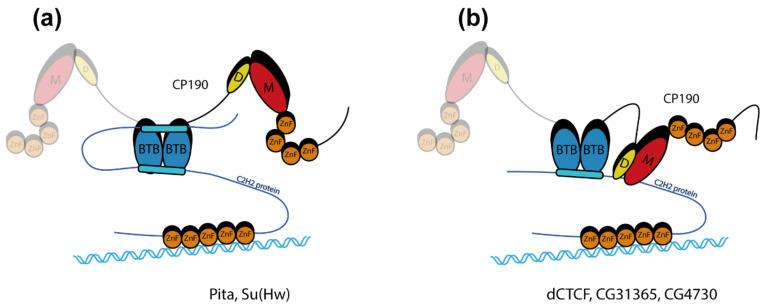
Schematic representation of models describing the specificity of CP190 interactions with C2H2 proteins. (**a**) The model of CP190 interaction with the Su(Hw) and Pita proteins, in which two regions additively interact with the BTB domain. (**b**) The model of CP190 interaction with proteins that interact simultaneously with the BTB and D/M domains of CP190 (dCTCF, CG31365, and CG4730). The second subunit of CP190 dimer except the BTB domain is shown as transparent.

In the pull-down assay, the interaction between Pita^220–232^ and the BTB was sensitive to all significant amino acid substitutions in the hydrophobic groove. In contrast, the interaction between the Pita^114–164^ region and the BTB is not sensitive to most of the BTB mutations. Since the Pita^∆220–232^ protein does not interact with CP190 in the Y2H assay, it seems likely that the Pita^114–164^ interaction with BTB is rather weak and uses only some of the potential contacts in the hydrophobic groove. It seems likely that Pita can form a stable complex with CP190 by simultaneous interaction of the key region Pita^220–232^ and the auxiliary region Pita^114–164^ with the BTB dimer (Figure 5a). A previous study [44] showed that two regions in the N-terminal part of Su(Hw) additively interact with the CP190 BTB domain. Here, we demonstrated that both Su(Hw) regions interact with comparable moderate affinity with the hydrophobic groove of the CP190 BTB. Our Y2H results are consistent with the model that both Su(Hw) regions additively interact with the BTB dimer in a similar manner as Pita (Figure 5a). 

Another mechanism of effective interaction with CP190 can be observed in the case of the dCTCF and CG31365 proteins (Figure 5b). For both of these proteins, only one region interacts with moderate affinity with the hydrophobic groove of the CP190 BTB. However, the relatively weak interaction with the BTB domain is compensated by additional interaction with the M domain of CP190. Thus, the high efficiency of the association of dCTCF or CG31365 with CP190 is achieved as a result of the additive interaction of two regions in C2H2 proteins simultaneously with the BTB and M domains of CP190.

The CG4730 protein has an unusual N-terminal unstructured region before the ZAD domain. This peptide cooperates with the ZAD to interact with the BTB domain of CP190. The CG4730 protein also interacts with the M and D domains of CP190. Thus, the strong association between CG4730 and CP190 is achieved through multiple simultaneous interactions. Interestingly, the N-terminal region in CG4730 is present only in orthologs from species closely related to *D. melanogaster*. It seems likely that in species evolutionarily distant from *D. melanogaster*, CG4730 interacts only with the M and D domains of CP190, as observed for Opbp and ZIPIC [35,36]. The existence of two domains in the architectural proteins that interact with CP190 allows the creation of an effective platform for recruitment of CP190 to chromatin sites.

Thus, C2H2 architectural proteins use several strategies to recruit CP190 protein. Most of them have two CP190-interacting sequences providing stability and redundancy of the interaction. The BTB domain of CP190 plays an important role in the binding to most architectural proteins via its hydrophobic grooves. Further studies are needed to elucidate the role of the hydrophobic groove in CP190 BTB in the recruitment of transcriptional complexes, as has been shown for the mammalian BCL6 and PLZF proteins.

## 4. Materials and Methods

### 4.1. Plasmid Construction

For in vitro experiments, protein fragments were either PCR-amplified using corresponding primers or digested from corresponding cDNAs and subcloned into pGEX-4T1 (GE Healthcare, Chicago, IL, USA) or into a vector derived from pACYC and pET28a(+) (Novagen) bearing a p15A replication origin and a kanamycin resistance gene. PCR-directed mutagenesis was used to make single amino acid substitutions in BTB CP190. The BTB domain of CP190 was cloned into a modified pET32a(+) vector containing TEV-cleavable thioredoxin and a 6×His-tag.

To express 3×FLAG-tagged proteins in the S2 cells, protein-coding sequences were subcloned into the pAc5.1 plasmid (Life Technologies, Carlsbad, CA, USA). Plasmids for the yeast two-hybrid assay were prepared using the full-sized and truncated versions of corresponding cDNAs fused with pGAD424 and pGBT9 vectors (Clontech, Mountain View, CA, USA). Details of the cloning procedures, primers, and plasmids used for plasmid construction are available upon request.

### 4.2. Yeast Two-Hybrid Assay

The yeast two-hybrid assay was performed as previously described [51]. Briefly, for growth assays, plasmids were transformed into yeast strain pJ69-4A by the lithium acetate method, following standard Clontech protocol, and plated on media without tryptophan and leucine. After two days of growth at 30 °C, the cells were plated on selective media without tryptophan, leucine, histidine, and adenine, and their growth was compared after 2–3 days. Each assay was repeated three times.

### 4.3. Protein Expression and Purification

BL21(DE3) cells transformed with a CP190^1–126^ construct fused with TEV-cleavable 6×His-thioredoxin were grown in 1 L of LB media to an A600 of 1.0 at 37 °C and then induced with 1 mM IPTG at 18 °C overnight. Cells were disrupted by sonication in buffer A (30 mM HEPES (pH 7.5), 400 mM NaCl, 5 mM β-mercaptoethanol, 5% glycerol, 0.1% NP40, 10 mM imidazole) containing 1 mM PMSF, and Calbiochem Complete Protease Inhibitor Cocktail VII (1 μL/mL). After centrifugation, lysate was applied to a Ni-NTA column, and, after washing with buffer B (30 mM HEPES (pH 7.5), 400 mM NaCl, 5 mM β-mercaptoethanol, 30 mM imidazole), was eluted with 300 mM imidazole. For cleavage of the 6×-His-thioredoxin-tag, 6×-His-tagged TEV protease was added at a molar ratio of 1:50 directly to the eluted protein and the mixture was incubated for 2 h at room temperature, then dialyzed against buffer A without NP-40 and applied to a Ni-NTA column. Flow-through was collected; dialyzed against 20 mM Tris-HCl (pH 7.4), 50 mM NaCl, and 1 mM DTT; and then applied to a SOURCE15Q 4.6/100 column (GE Healthcare, USA) and eluted with a 50–500 mM linear gradient of NaCl. Fractions containing protein were concentrated, frozen in liquid nitrogen, and stored at −70 °C.

### 4.4. Protein Crystallization, Data Collection and Processing, and Structure Solution

The BTB domain of CP190 was crystallized using a counter-diffusion technique in glass capillaries [57], using crystallizations conditions obtained previously by the vapor diffusion technique. A microgravity experiment was conducted on the International Space Station [58] to decrease convection [59], which allowed us to obtain high-quality protein crystals. Crystals were grown at +20 °C in the following conditions: 0.1 M HEPES (pH 7.5), 0.8 M ammonium phosphate monobasic, 0.8 potassium phosphate monobasic.

The X-ray dataset was collected at 100 K to a resolution of 1.4 Å using a Pilatus 6M-F detector on the BL41XU beamline at the SPring-8 synchrotron-radiation facility (Japan). Crystals were briefly soaked in 25% (*v*/*v*) glycerol for cryoprotection. The following data collection strategy was predicted by HKL2000 [60]: wavelength, 1.0 Å; rotation angle, 80°; oscillation angle, 0.5°; crystal to detector distance, 240 mm. Data was processed with Mosflm [61]. Data collection and refinement statistics are summarized in Supplementary Materials Table S4.

Structure solution was performed using the molecular replacement method with the Molrep program [62] using a known structure of the CP190 BTB domain (PDB code 4U77) as a starting model.

Structure refinement was performed using Refmac5 [63] and COOT [64] programs. There is one protein monomer in the asymmetric unit. The resolution was gradually increased to 1.4 Å during refinement. Hydrogens in rigid positions as well as anisotropic B-factor refinement were used in the last stages of the refinement. The final model comprises one subunit of the protein (121 residues), 125 water molecules, and a phosphate molecule. The first two N-terminal residues as well as three C-terminal residues were not observed in electron density maps, due apparently to the high mobility of these residues. The structure was verified with the Molprobity [65] and PDB_REDO [66] servers. The visual inspection of the structure model was carried out with COOT and PyMOL (The PyMOL Molecular Graphics System, Schrödinger, LLC, USA). Comparison of the structures was made with the PDBeFOLD program [67]. The contacts were analyzed using the PDBePISA [68] and WHATIF servers [69]. The free energy of solvation upon the formation of the dimer was estimated with PDBePISA [68]. Structural data were deposited to the Protein Data Bank (www.rcsb.org) on 16 October 2017 under accession code 6ER1. Molecular graphics were prepared using UCSF Chimera [70].

### 4.5. Pull-Down Assays and Chemical Crosslinking

GST-pull-down was performed with Immobilized Glutathione Agarose (Pierce, Lakewood, WA, USA) in buffer C (20 mM Tris (pH 7.5); 150 mM NaCl; 10 mM MgCl_2_; 0.1 mM ZnCl_2_; 0.1% NP40; 10% [*w*/*w*] glycerol; 1 mM DTT). BL21 cells co-transformed with plasmids expressing GST-fused derivatives of C2H2 proteins and 6×His-Thioredoxin-fused CP190^1–126^ or CP190^245–606^ were grown in LB media to an A600 of 1.0 at 37 °C and then induced with 1 mM IPTG at 18 °C overnight. ZnCl_2_ was added to a final concentration of 100 μM before induction. Cells were disrupted by sonication in 1 mL of buffer C, and after centrifugation, lysate was applied to pre-equilibrated resin for 10 min at +4 °C; subsequently, resin was washed four times with 1 mL of buffer C containing 500 mM NaCl and bound proteins were eluted with 50 mM reduced glutathione, 100 mM Tris (pH 8.0), and 100 mM NaCl for 15 min. 6×His-pull-down was performed similarly, with Ni-NTA HP resin (GE Healthcare, USA) in buffer A (see the protein expression and purification section) containing 1 mM PMSF and Calbiochem Complete Protease Inhibitor Cocktail VII (5 μL/mL) and washed with buffer A containing 30 mM imidazole. Proteins were eluted with buffer B containing 300 mM imidazole (20 min at +4 °C). Chemical crosslinking was carried out for 10 min at room temperature in 20 mM HEPES (pH 7.7); 150 mM NaCl; 20 mM imidazole; and 1 mM β-mercaptoethanol. Prior to crosslinking, protein concentration was adjusted to 5 μM for at least 1 h. Crosslinking was quenched with 50 mM Tris-HCl (pH 6.8), and samples were resolved using SDS-PAGE followed by silver staining.

### 4.6. Co-Immunoprecipitation Assay

Drosophila S2 cells were grown in SFX medium (HyClone, Logan, UT, USA) at 25 °C. S2 cells grown in SFX medium were co-transfected with 3×FLAG-CG4730 or 3×FLAG-CG31365 and CP190 plasmids with Cellfectin II (Life Technologies, USA) as recommended by the manufacturer. Protein extraction and co-immunoprecipitation were performed as described in [51]. Anti-FLAG antibodies (clone M2, Sigma Aldrich, St. Louis, MI, USA) and mouse IgG were used for co-immunoprecipitations. The results were analyzed by Western blotting. Proteins were detected using the ECL Plus Western Blotting substrate (Pierce, USA) with anti-FLAG (Sigma Aldrich, USA), anti-CP190 (produced in rats against recombinant protein), and anti-lamin (clone ADL84.12, DSHB, University of Iowa) antibodies.

## 5. Conclusions

In this study, we found that architectural proteins use various mechanisms to improve the efficiency of interaction with CP190. Pita and Su(Hw) have two regions that appear to simultaneously interact with hydrophobic grooves in the BTB dimer. dCTCF and the newly identified CG31365 have two adjacent regions that interact simultaneously with the hydrophobic groove of the BTB and the M domain of CP190. Another protein described for the first time here, CG4730, interacts with the BTB, M, and D domains of CP190 simultaneously. 

## Data Availability

All data generated or analyzed during this study are included in this published article and its Supplementary Information files. Structural data were deposited to the Protein Data Bank (www.rcsb.org) under the accession code 6ER1.

## Supplementary Materials

The following are available online at https://www.mdpi.com/article/10.3390/ijms222212400/s1.
