# Supplementary material for: Mechanisms of CP190 Interaction with Architectural Proteins in Drosophila Melanogaster
Source: Int J Mol Sci. 2021 Nov 17;22(22):12400. doi: 10.3390/ijms222212400 (PMC8618245; doi:10.3390/ijms222212400)
Supplement: Supplementary file 1 [file ijms-22-12400-s001.zip › ijms-1463203-supplementary.pdf]

Supplementary information for

Mechanisms of CP190 interaction with architectural proteins in *Drosophila melanogaster*

Marat Sabirov[1,2], Anastasia Popovich[1], Konstantin Boyko[1,3], Alena Nikolaeva[4], Olga Kyrchanova[1,2], Oksana Maksimenko[2], Vladimir Popov[3,4], Pavel Georgiev[1*], Artem Bonchuk[1,2*]

[1] Department of the Control of Genetic Processes, Institute of Gene Biology Russian Academy of Sciences, 34/5 Vavilov St., Moscow 119334, Russia; greencapers@yandex.ru (M.S.), annafedotova@list.ru (A.F.), anast.popovich@gmail.com (A.P.), olgina73@gmail.com (O.K.)

[2] Center for Precision Genome Editing and Genetic Technologies for Biomedicine, Institute of Gene Biology, Russian Academy of Sciences, 34/5 Vavilov St., Moscow 119334, Russia; maksog@mail.ru (O.M.)

[3] Research Center of Biotechnology, Russian Academy of Sciences, Leninsky pr-t, 33, bld. 2, Moscow 119071, Russia; boiko_konstantin@inbi.ras.ru (K.B.), vpopov@inbi.ras.ru (V.P.)

[4] National Research Center "Kurchatov Institute", Moscow, Russia; aishome@mail.ru (A.N.)

* Correspondence to Artem Bonchuk (A.B.) and Pavel Georgiev (P.G.): georgiev_p@mail.ru (P.G.) bonchuk_a@genebiology.ru (A.B.)

Supplementary Data

Detailed description of CP190BTB domain structure

The crystal structure of CP190 BTB-domain (hrBTB) was refined to 1.4Å resolution. Despite of the best obtained resolution among other known structures of *D.melanogaster* BTB-domain of CP190 (codes 4U77 and 5EUP), new structure demonstrate no new important structural features. RMSD between Cα-atoms upon superposition varies from 0.4 to 0.7 $Å^2$ for 4U77 and 5EUP, accordingly. Contact analysis made with PDBePISA showed that BTB-domain exists in a homodimeric state in a crystal, which is in agreement with data obtained earlier [1,2].

Comparison of dimerization interface for known CP190 BTB dimers revealed some differences. Firstly, Lys19 in case of hrBTB tends to have two conformations: one similar to 4U77 structure, where Lys7 in involved in dimerization interface by forming hydrogen bond to main-chain carboxyl of Ala51 of adjacent subunit, and other - similar to 5EUP, where Lys7 is not involved in dimerization. Secondly, side chain of His122 in structure of hrBTB, which is clearly seen in electron density, occupies completely different position compared to 4U77 (this residue is invisible in case of 5EUP). This unique position could strengthen the dimeric interface via its stacking interactions to side-chain of Tyr29 of adjacent subunit.

Supplementary Tables

Table S1. C2H2 proteins used in the Y2H screen.

Annotation Symbol	Name	pGBT	$CP190^{BTB}$
CG10031	Bowl	-	-
CG12653	btd	-	-
CG8367	Cg	-	-
CG1024	*unnamed*	-	-
CG1130	*unnamed*	-	-
CG1233	*unnamed*	-	-
CG1244	*unnamed*	-	-
CG1322	*unnamed*	-	-
CG1343	*unnamed*	-	-
CG1529	*unnamed*	-	-
CG1603	*unnamed*	-	-
CG1663	*unnamed*	-	-
CG1792	*unnamed*	-	-
CG1832	*unnamed*	-	-
CG2052	*unnamed*	-	-
CG2102	*unnamed*	-	-
CG2116	*unnamed*	-	-
CG2120	*unnamed*	-	-
CG2129	*unnamed*	-	-
CG2199	*unnamed*	-	-

Annotation Symbol	Name	pGBT	$CP190^{BTB}$
CG10654	*unnamed*	-	-
CG10959	*unnamed*	-	-
CG11247	*unnamed*	-	-
CG11398	*unnamed*	-	-
CG11414	*unnamed*	-	-
CG11695	*unnamed*	-	-
CG11696	*unnamed*	-	-
CG11906	*unnamed*	-	-
CG11924	Cf2	-	-
CG12029	*unnamed*	-	-
CG12260	*unnamed*	-	-
CG12299	*unnamed*	-	-
CG12391	*unnamed*	-	-
CG12605	*unnamed*	-	-
CG12744	*unnamed*	-	-
CG12769	*unnamed*	-	-
CG12942	*unnamed*	-	-
CG14438	*unnamed*	-	-
CG14451	*unnamed*	-	-
CG14655	*unnamed*	-	-

CG2678	*unnamed*	-	-
CG2712	*unnamed*	-	-
CG2889	*unnamed*	-	-
CG2932	bteb2	-	-
CG3065	*unnamed*	-	-
CG3281	*unnamed*	-	-
CG3445	*unnamed*	-	-
CG3526	*unnamed*	-	-
CG3758	esg	-	-
CG3847	*unnamed*	-	-
CG4158	*unnamed*	-	-
CG4360	*unnamed*	-	-
CG4424	*unnamed*	-	-
CG4427	*unnamed*	-	-
CG4456	*unnamed*	-	-
CG4496	*unnamed*	-	-
CG4677	*unnamed*	-	-
CG4694	*unnamed*	-	-
CG4707	*unnamed*	-	-
**CG4730**	***unnamed***	**-**	+
CG4820	*unnamed*	-	-
CG4854	*unnamed*	-	-
CG4936	*unnamed*	-	-
CG5245	*unnamed*	-	-
CG5249	*unnamed*	-	-
CG5557	*unnamed*	-	-
CG5669	*unnamed*	-	-
CG6654	*unnamed*	-	-
CG6689	*unnamed*	-	-
CG6791	*unnamed*	-	-
CG6808	*unnamed*	-	-
CG7101	*unnamed*	-	-
CG7204	Neu2	-	-
CG7368	*unnamed*	-	-
CG7672	*unnamed*	-	-
CG7691	*unnamed*	-	-
CG7928	*unnamed*	-	-
CG7963	*unnamed*	-	-
CG7987	*unnamed*	-	-
CG8089	*unnamed*	-	-
CG8145	*unnamed*	-	-
CG8159	*unnamed*	-	-
CG8319	*unnamed*	-	-
CG8388	*unnamed*	-	-
CG8474	*unnamed*	-	-
CG8643	*unnamed*	-	-
CG9167	*unnamed*	-	-
CG9215	*unnamed*	-	-
CG9650	*unnamed*	-	-
CG9793	*unnamed*	-	-
CG14667	*unnamed*	-	-
CG14962	*unnamed*	-	-
CG15073	*unnamed*	-	-
CG15336	*unnamed*	-	-
CG16779	*unnamed*	-	-
CG17181	*unnamed*	-	-
CG17328	*unnamed*	-	-
CG17385	*unnamed*	-	-
CG17508	*unnamed*	-	-
CG17612	*unnamed*	-	-
CG17743	pho	-	-
CG17803	*unnamed*	-	-
CG17806	*unnamed*	-	-
CG17829	*unnamed*	-	-
CG18011	*unnamed*	-	-
CG18262	*unnamed*	-	-
CG18476	*unnamed*	-	-
CG30020	*unnamed*	-	-
CG30431	*unnamed*	-	-
**CG31365**	***unnamed***	**-**	+
CG31612	*unnamed*	-	-
CG31632	*unnamed*	-	-
CG31670	*unnamed*	-	-
CG31782	*unnamed*	-	-
CG32120	*unnamed*	-	-
CG32767	*unnamed*	-	-
CG32772	*unnamed*	-	-
CG33221	*unnamed*	-	-
CG2125	ci	-	-
CG14938	crol	-	-
**CG8591**	**CTCF**	**-**	+
CG10269	D19A	-	-
CG10270	D19B	-	-
CG2711	dwg	-	-
CG9233	Fu2	-	-
CG33133	Grau	-	-
CG8436	Ibf1	-	-
CG9740	Ibf2	-	-
CG11352	jim	-	-
CG9397	jing	-	-
CG3340	Kruppel	-	-
CG33473	Luna	-	-
CG9797	M1BP	-	-
CG3743	MTF	self-activation	
CG12809	nerfin-2	-	-
CG3851	Odd	-	-
CG1133	opa	-	-
CG30443	Opbp	-	-
**CG3941**	**Pita**	**-**	+
CG7752	Z4	-	-

CG9932	*unnamed*	-	-	CG8092	Row	-	-
CG10267	*unnamed*	-	-	CG3956	snail	-	-
CG10321	*unnamed*	-	-	CG3242	Sob	-	-
CG10462	*unnamed*	-	-	CG7847	Sr	-	-
CG10543	*unnamed*	-	-	CG7938	Sry-beta	-	-
CG10654	*unnamed*	-	-	**CG8573**	**Su(Hw)**	**-**	+
CG8484	Topi	-	-	CG3998	Zf30c	-	-
CG4148	Wek	-	-				

Table S2. Peptides derived from Pita and CTCF proteins used for crystallization trials in complex with CP190 BTB:

Pita[215-240]	SGSPVKPKVLNKSSIRILNKGPAAPVEP
Pita[215-232]	SGSPVKPKVLNKSSIRILNKY
Pita[220-232]	SGSVLNKSSIRILNKY
CTCF[715-733]	SGSMIINQRLRSQRGTKTFHIKY

Table S3. Oligonucleotides used for cloning. Restriction enzyme sites are shown in small letters, the corresponding enzymes are noted. Nucleotide substitutions in mutagenic primers are also shown in small letters.

Name	Sequence	restriction enzyme
CTCF733Yr	gatctcgagttaataGgGCTTGATGTGGAACGTC	*Xho*I
CP190F15Ad	GGGAGTCgcCTTCCTGCAGAAG	
CP190F15Ar	CTGCAGGAAGgcGACTCCCCAG	
CP190K19Ad	CTTCCTGCAGgcGCTGCAGAAC	
CP190K19Ar	GTTCTGCAGCgcCTGCAGGAAG	
CP190K117S	GTGCTGTTGtcGCTGCTAGAGGC	
CP190K117r	CTCTAGCAGCgaCAACAGCACC	
CP190V7A	CAAGTCCGcGAAAGTGGACAAC	
CP190V7Ar	GTCCACTTTCgCGGACTTGAC	
CP_L118A	GTTGAAGgcGCTAGAGGCGC	

CP_L118Ar	CTCTAGCgcCTTCAACAGCAC	
CP190V7H	CAAGTCCcatAAAGTGGACAAC	
CP190V7Hr	GTCCACTTTatgGGACTTGACTTC	
CP190V114N	CATGACGaatCTGTTGAAGC	
CP_V114Nr	GCTTCAACAGattCGTCATGTTC	
CP190_212r	ttgactaGTTGGCTCCTGCTTCACATTG	*Spe*I
CP190_298d	aagactagtTCCCCACAGGGTACACAG	*Spe*I
CP190_308r	tgtactagtATGTTCTAGTTGGGTCTGTG	*Spe*I
CP190_374d	ttGACtagtAAATCTGCTCCAGCGACAG	*Spe*I
CP190_204d	aatagatctAATGTGAAGCAGGAGCCAAC	BglII
CP190_373r	ttactcgagCGGGGTATTGGGCATAATC	*Xho*I
CP190_470r	tagctcgAGttAGGGCCCAGTAGTATTCTCTG	*Xho*I
CP190V114A	CATGACGGcGCTGTTGAAGC	
CP_V114Ar	GCTTCAACAGCgCCGTCATG	
SuHw98d	GCTggaTCCACTGTGAAGATTCTG	*Bam*HI
Su(Hw)187r	ATCGtcgacttaCTCGGTGACAACGATC	*Sal*I
CG4730[1]d	AAgaattcATGGATCCCCAAACATCAAA	EcoRI
4730_1d	TTTgaattcTGGATCCCCAAACATCAAA	*Eco*RI
PD4730D_128	TTTgaattcCAACCGAAGCCAACACG	*Eco*RI
4730R44	ttgtcgacATTGCTCAGCTCCAAAGA	*Sal*I
4730R_133	ttGTCGAcCGTGTTGGCTTCGGTT	*Sal*I
4730[180]r	ATgtcgacGATTTTCCCGTATGCCGATT	*Sal*I
4730[360]r	ATgtcgacAACTTTCGCATTTCCTCCA	*Sal*I
4730D45	tttgaattcAATTGCTGCCGCTTGT	*Eco*RI
PD4730D45	ttggatccAATTGCTGCCGCTTGT	*Bam*HI
PD4730R131	ttctcgagctaGGCTTCGGTTGCCACAAA	*Xho*I
ip4730r	ttctcgagTTATTTAGAAATAGCAGCGCT	*Xho*I
4730R348	ttgtcgacCATGTTATGCTTACGCTCCA	*Sal*I
4730D346	tttgaattcAACATGGTGGTGCAGATG	*Eco*RI

4730R392	ttgtcgacTTTAGAAATAGCAGCGCTATCAT	*Sal*I
31365D_4	tctaCtgcaGCACAGCCGTCGTATCCCGATTT	*Pst*I
31365D_108	ttggatccGACGAGGACGAGCTGGAGTT	*Bam*HI
31365D_226	TTTggatccTGGGAGTCTTTGGATGGGTC	*Bam*HI
31365D_368	tttggatcccagttcagtggcgaaaagcc	*Bam*HI
31365R_103	TCTTagatctGATCCTTGGAGAAGACGCGG	*Bgl*II
31365R_455	tctaAgatctCACAAATGGCACTGGAACGA	*Bgl*II
PD31365R_455	ttctcgagCACAAATGGCACTGGAACGA	*Xho*I
31365R_453	TTcggccgCACAAATGGCACTGGAACGA	*Eag*I
31365R_end	TTgtcgacCTAGGCCACAGCTTGAAACTC	*Sal*I
31365_271d	tttGGATCCGCCAGGAAGGATGCAAAAGA	*Bam*HI
31365_324d	tttGGATCCGTGCACACAGACAATGGAGAG	*Bam*HI
31365_380D	tttGGATCCGTGGTCGTGTTTAATCTGGGC	*Bam*HI
31365_350r	ttctcgagAAATTCGGGAGTGGAGTCCTG	*Xho*I
31365_400r	ttctcgagTTCATGGAAGCTGAAGACCTG	*Xho*I
31365_420r	ttctcgagCTAataTCGTTTGAGGGGCGTCTG	*Xho*I
ip31365d	ttgcggccgcGGATGCACAGCCGTCG	*Not*I
ip31365r	ttgggcccctaggCCACAGCTTGAAAC	*Avr*II
31365R200	ttagatctCTCGCTCACCTCGTTTCGCA	*Bgl*II
31365R230	ttagatctATCCAAAGACTCCCACCGACC	*Bgl*II
31365R303	ttagatctCGTCCAGTTCGAGGCCATCTA	*Bgl*II

Table S4. Data collection and refinement statistics of CP190 BTB-domain.

**Data collection**	
Space group	$P3_221$
Cell dimensions	
a, b, c (Å)	84.94 84.94 40.40
α, β, γ (°)	90; 90; 120
Resolution (Å)	73.560 -1.40 (1.42-1.40)
R_{meas} (%)	5.0 (66.4)
$CC_{1/2}$	99.7 (81.6)
<I>/<σ(I)>	15.5 (2.6)
Completeness	98.2 (99.9)
Redundancy	4.1 (4.2)
**Refinement**	
R_{work} / $R_{free.}$	14.6 / 18.1
No. of atoms	
Protein	1015
Ligans/ion	5
Water	125
Ramachandran outliers, %	0
Ramachandran favored, %	99.16
R.m.s deviations	
Bond length (Å)	0.018
Bond angle (°)	1.910
MolProbity Score	1,28

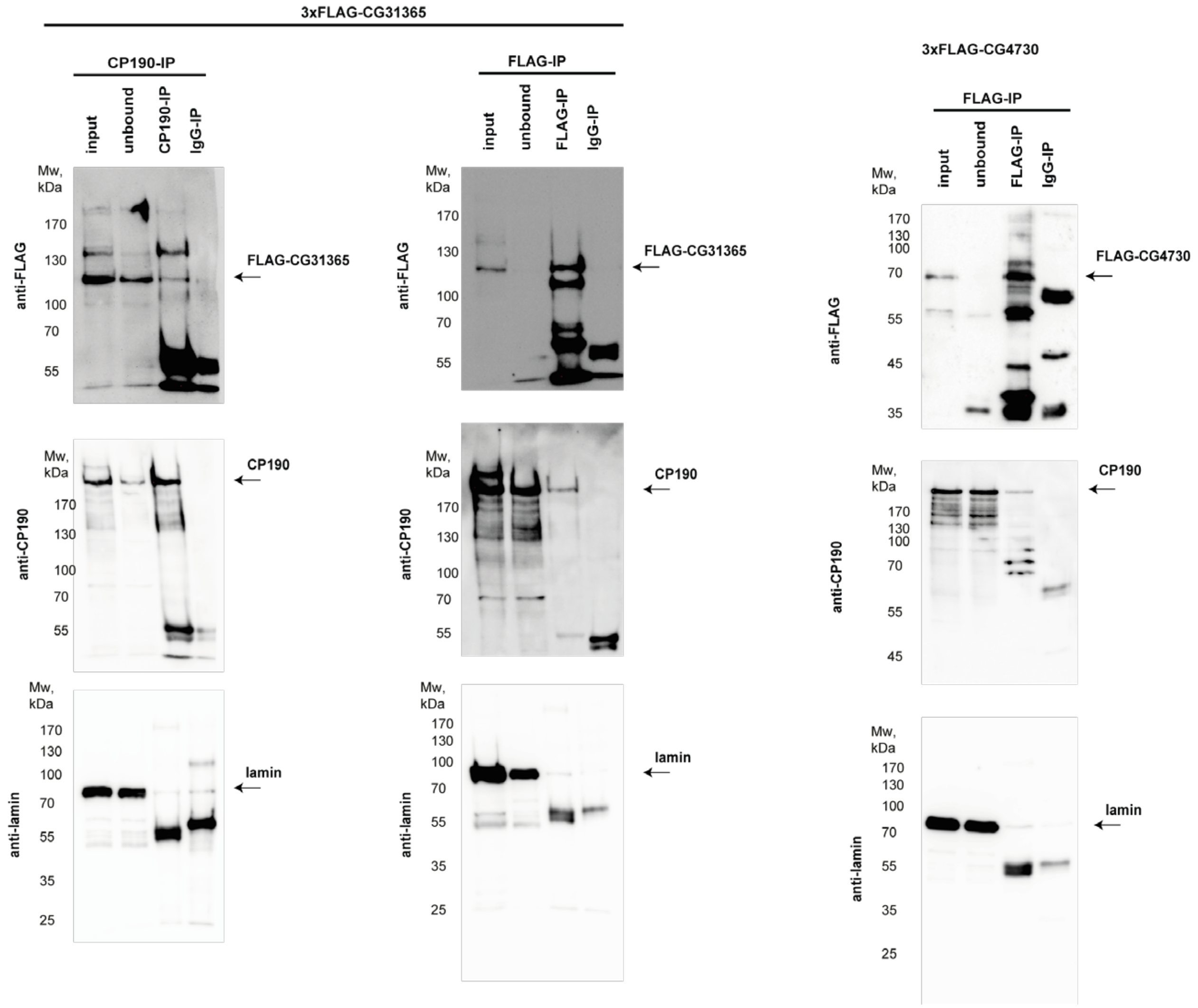

Figure S1. Co-immunoprecipitation of CG4730 or CG31365 tagged with FLAG and CP190 from S2 cell extracts. The FLAG-CG4730 and FLAG- CG31365 were expressed in S2 cells. Total extracts immunoprecipitated with antibodies against CP190 or FLAG. The immunoprecipitates (IP) were analyzed by Western blotting.

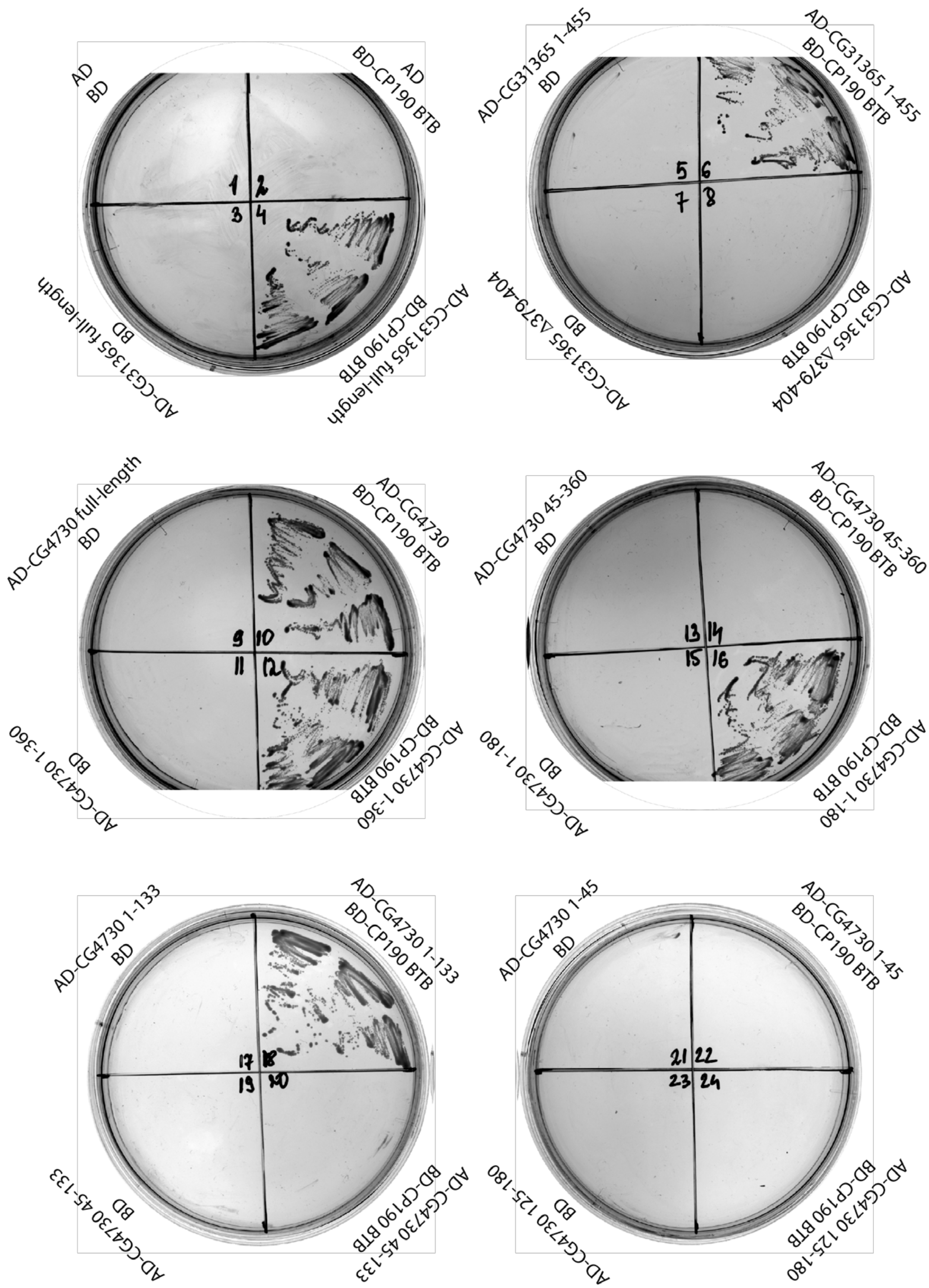

Figure S2. Testing the interactions between C2H2 proteins and CP190 using yeast two-hybrid assay. Growth assay plates without histidine are shown (yeasts are unable to grow on this medium in the absence of interaction). AD stands for Activation Domain, BD – for DNA-Binding Domain of GAL4 protein.

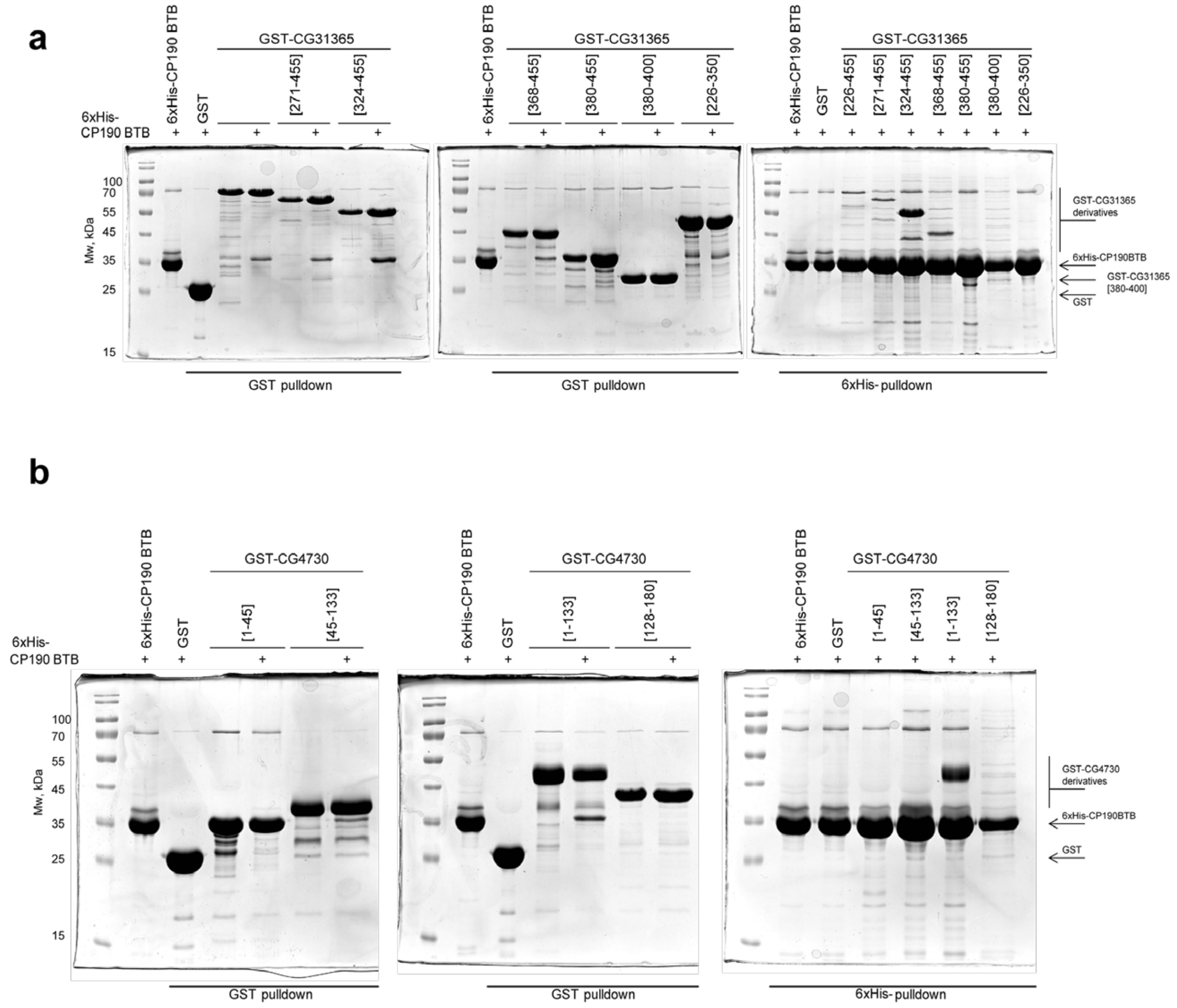

Figure S3. Results of testing the interaction between bacterially expressed 6x-His-tagged CP190 BTB domain and GST-tagged deletion derivatives of CG4730 and CG31365 proteins in GST- and 6xHis-pulldown assays. Position of CP190 BTB-domain is shown with asterisk.

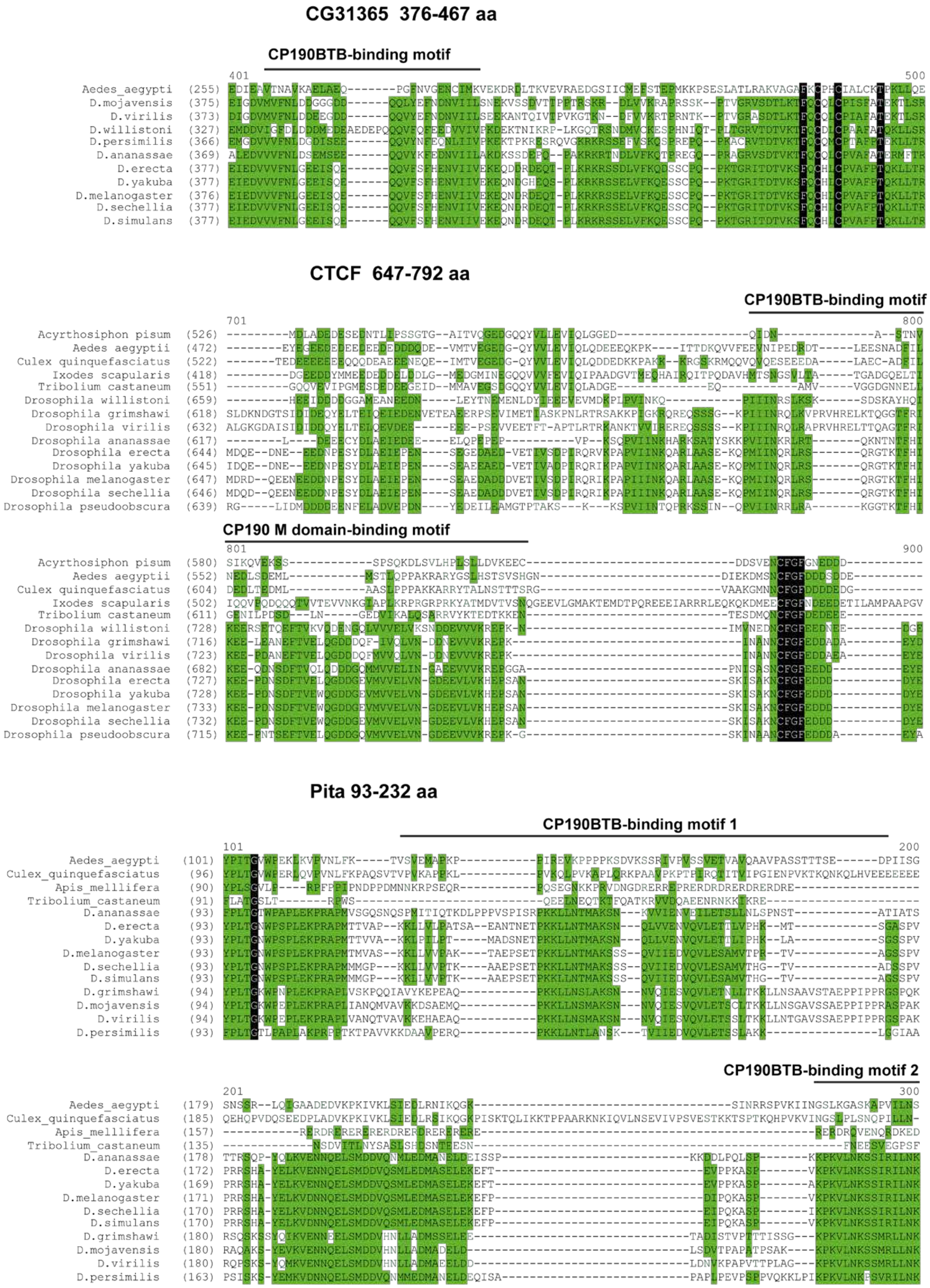
CG31365 376-467 aa
CP190BTB-binding motif
CTCF 647-792 aa
CP190BTB-binding motif
CP190 M domain-binding motif
Pita 93-232 aa
CP190BTB-binding motif 1
CP190BTB-binding motif 2

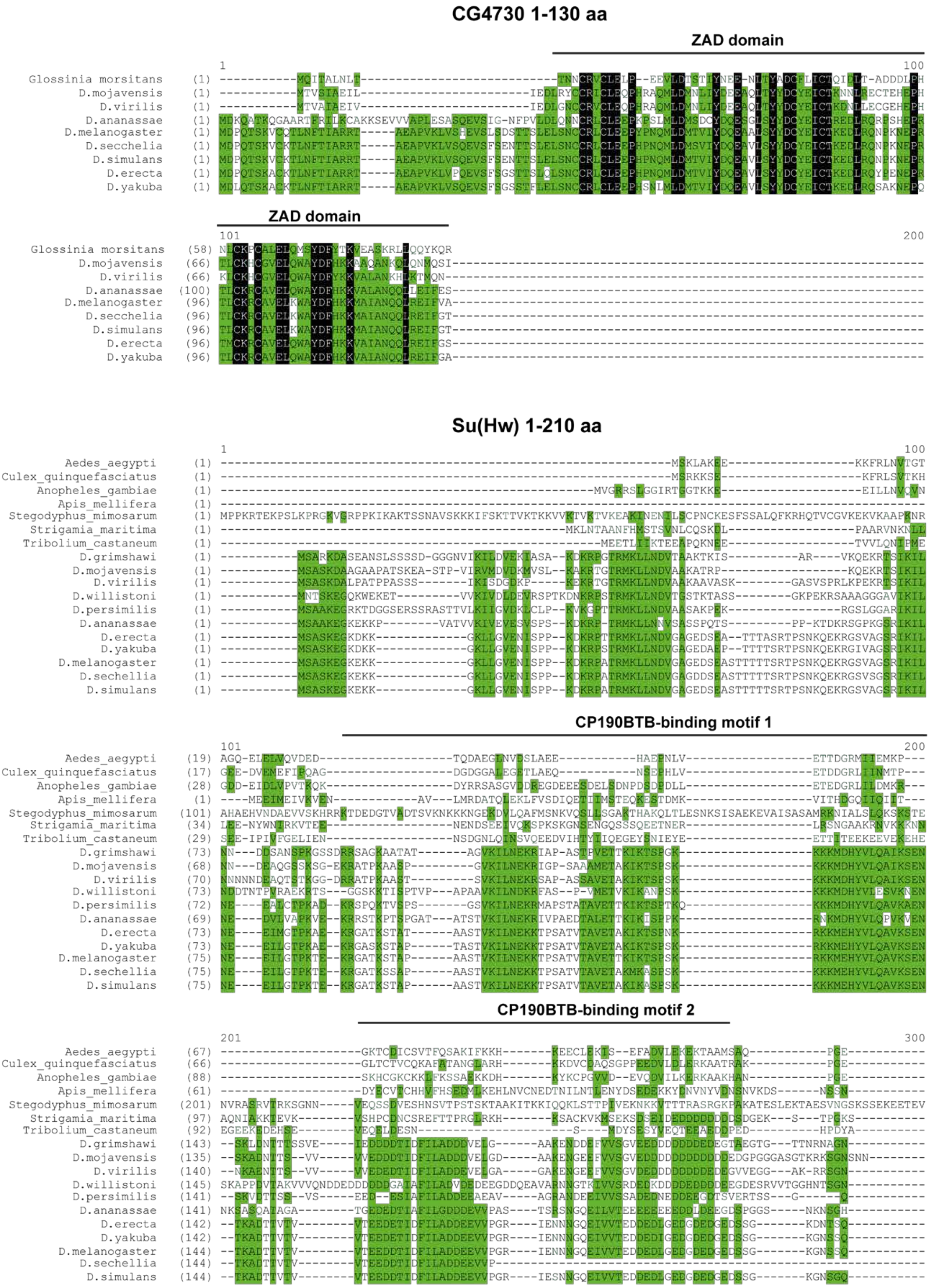

Figure S4. Conservation of the BTB-binding sequences in C2H2 proteins from various insects. The residue numbering is according to the *D. melanogaster* protein. Conserved residues are shown in green, identical – in black.

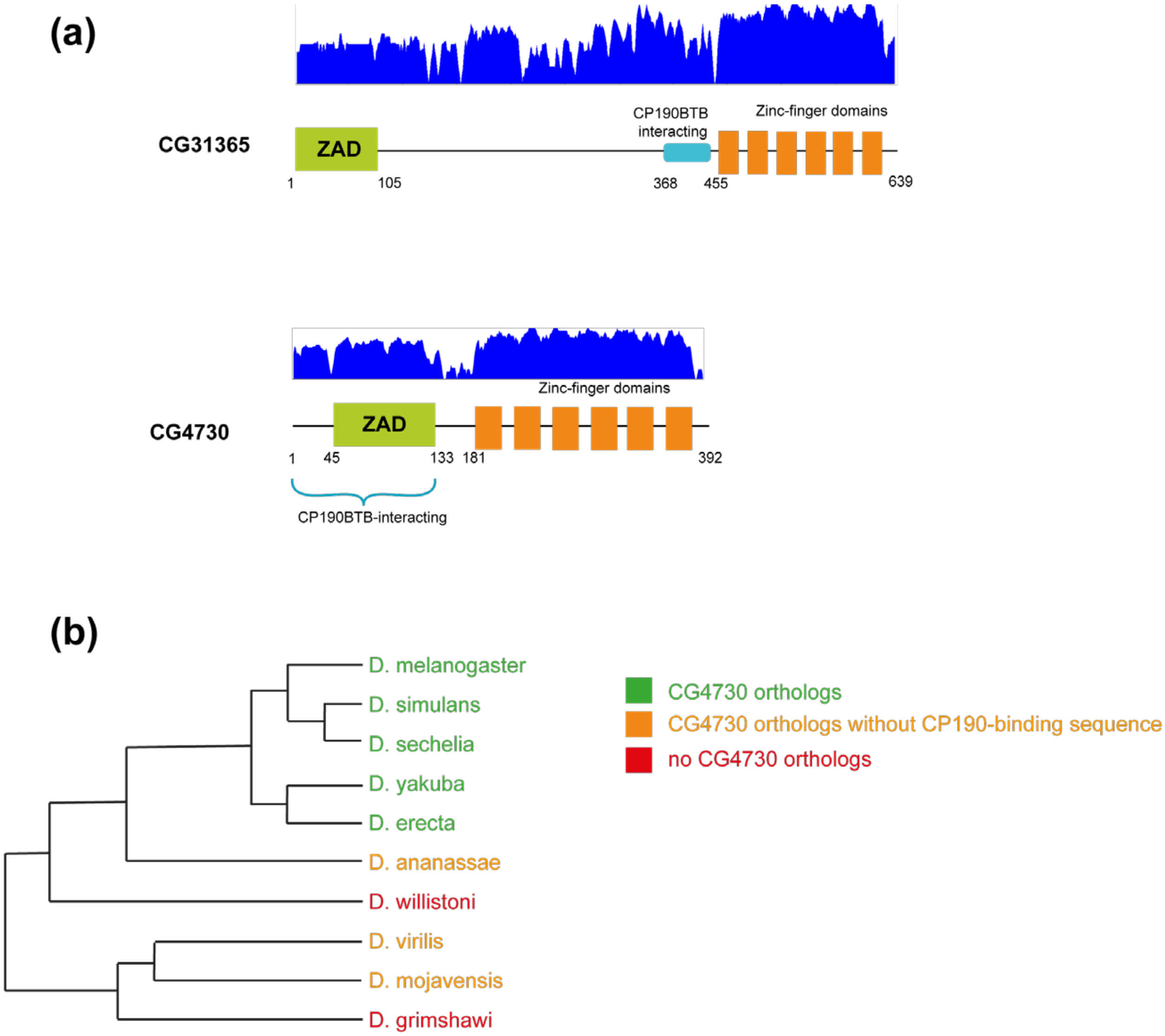

Figure S5. (a) The profiles of CG4730 and CG31365 sequence conservation across *Drosophila* species. **(b)** The presence of CP190-binding sequence in CG4730 orthologs shown at the phylogenetic tree of Drosophila genus. The tree is adapted from [3].

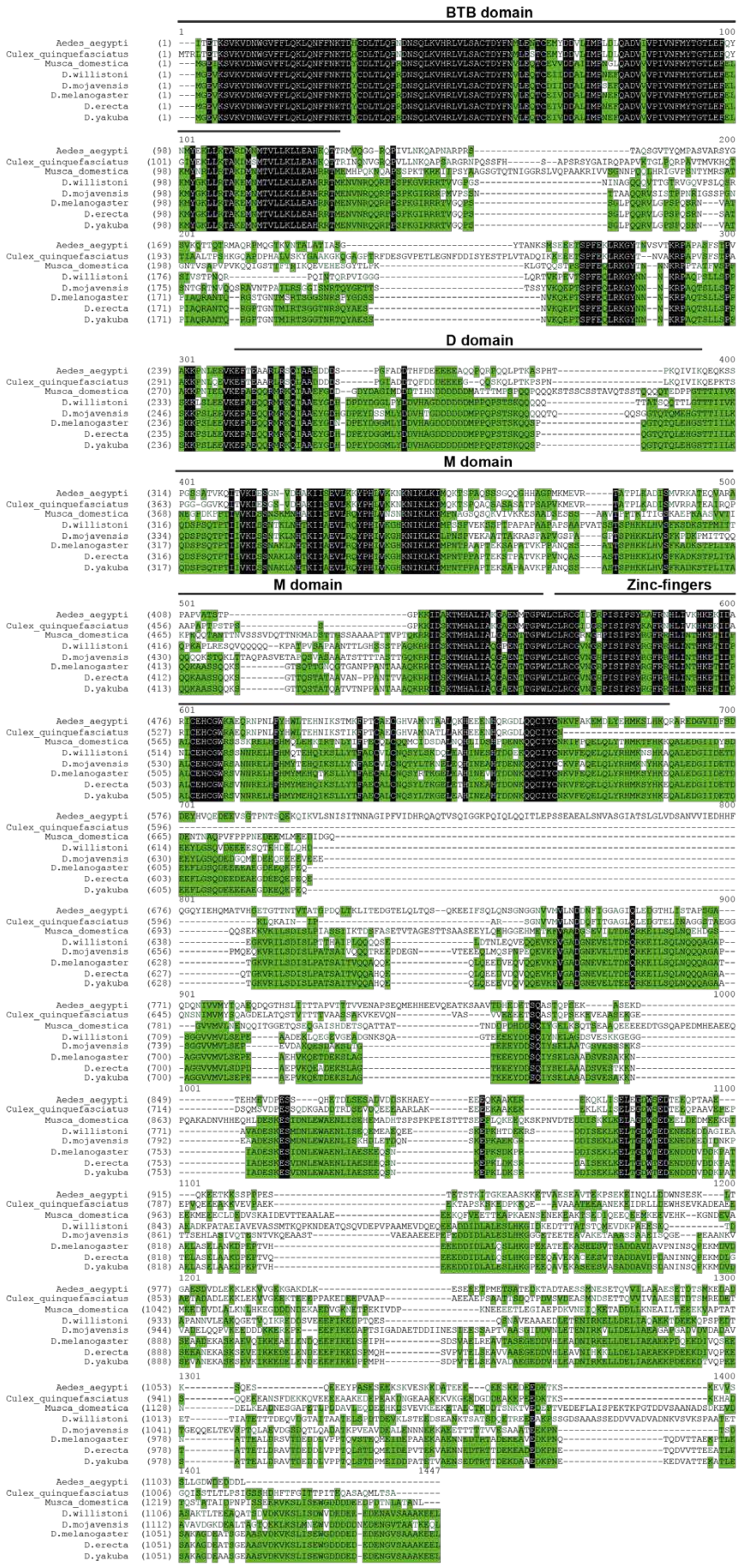

Figure S6. Multiple sequence alignment of CP190 proteins from various insects. The residue numbering is according to the *D. melanogaster* protein. Color scheme is the same as in Figure S4.

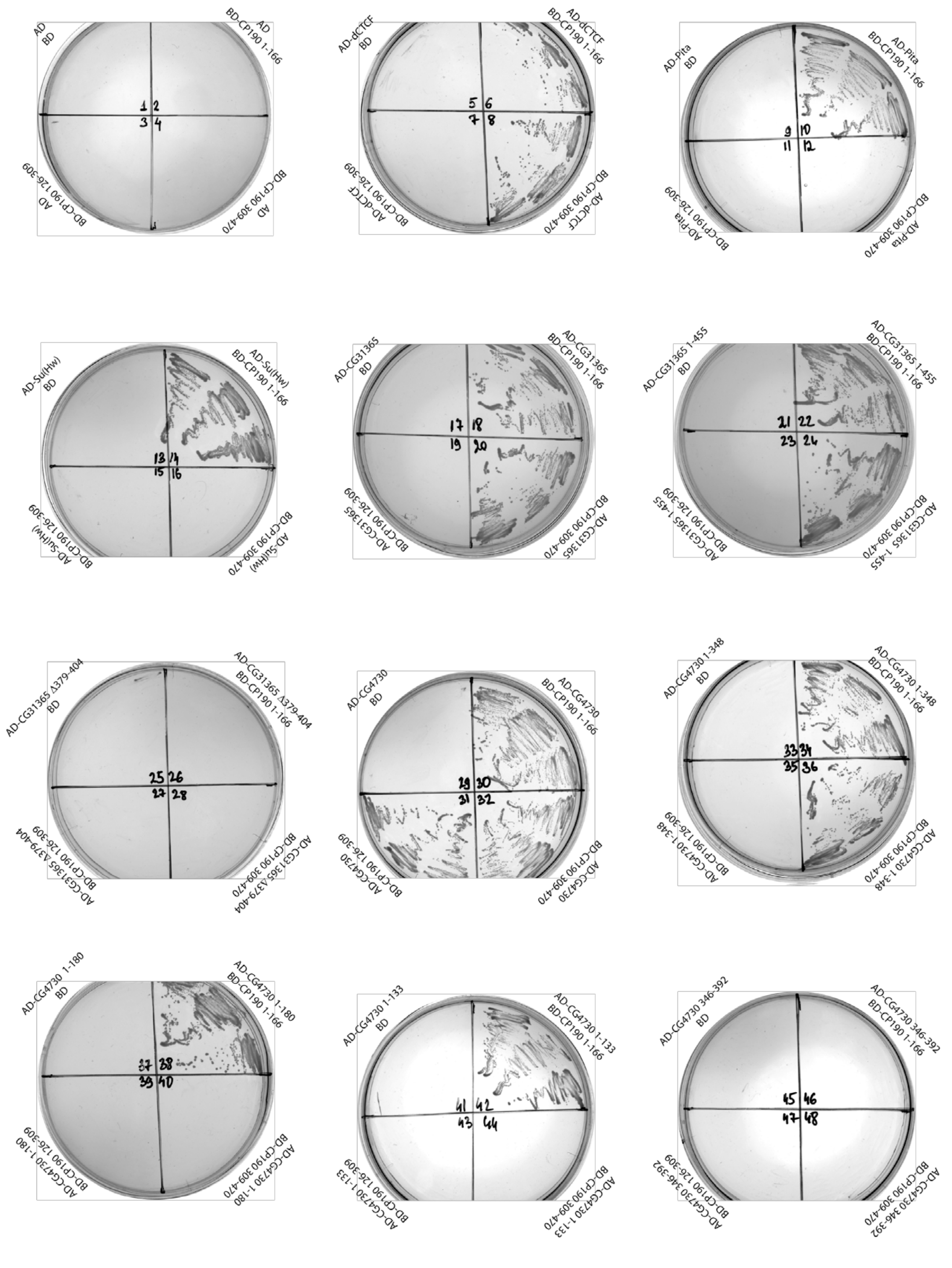

Figure S7. Testing the interactions between C2H2 proteins and CP190 deletion derivatives using yeast two-hybrid assay. Growth assay plates without histidine are shown (yeasts are unable to grow on this medium in the absence of interaction). AD stands for Activation Domain, BD – for DNA-Binding Domain of GAL4 protein.

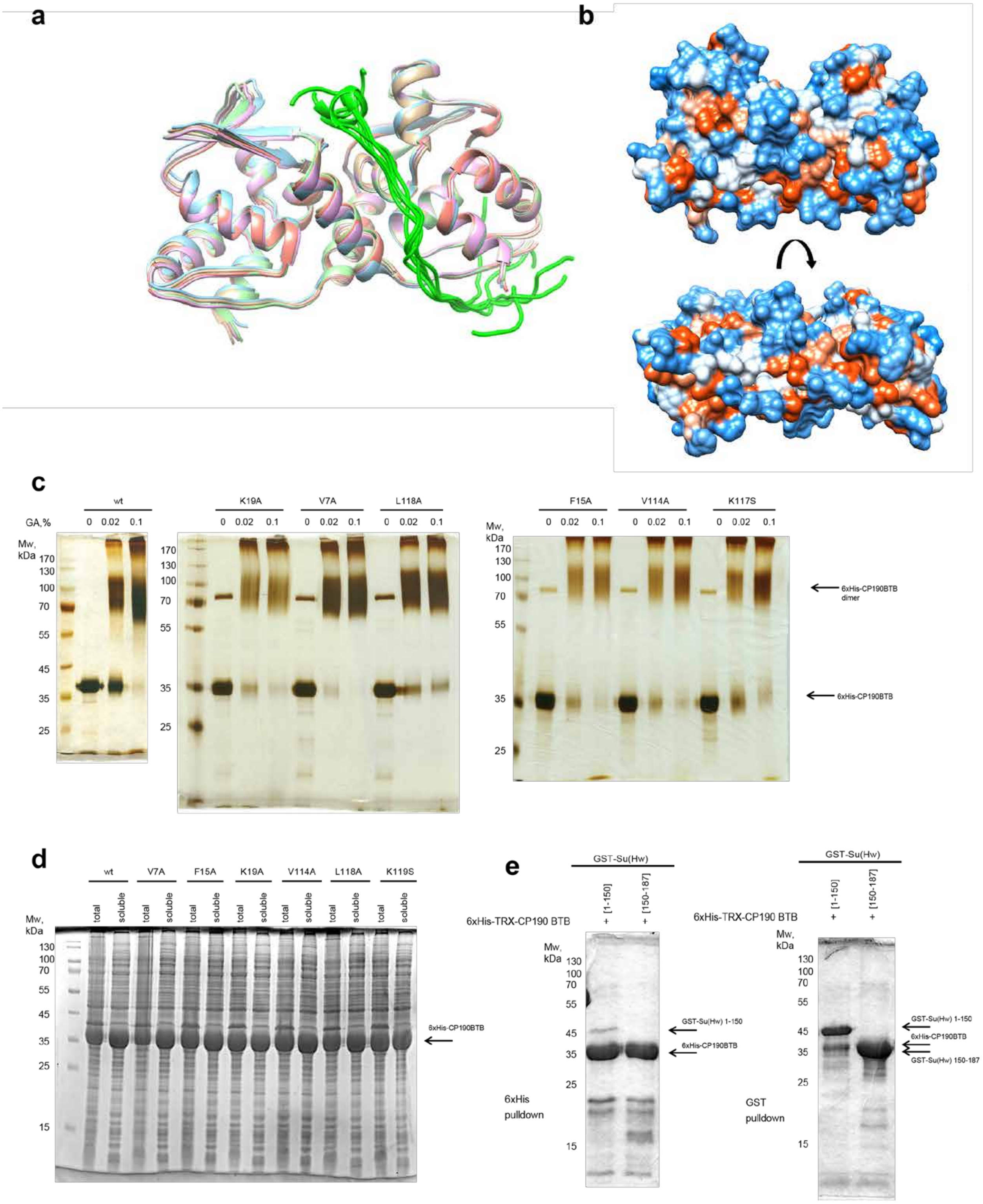

Figure S8. **(a)** Superposition of semi-flexible docking models of Pita peptide 220-232 (shown in green) and the crystal structure of CP190. **(b)** Hydrophobic surface of CP190 BTB domain. **(c)** Chemical crosslinking of Thioredoxin-tagged CP190 BTB-domain bearing single amino-acid substitutions using increased concentrations of Glutaraldehyde (GA, indicated above). Molecular weight markers are shown. **(d)** Testing of solubility of bacterially expressed BTB-domain mutants. **(e)** Analysis of the interaction of Su(Hw) N-terminal domain with CP190 BTB-domain using pull-down assay *in vitro* with bacterially expressed proteins.

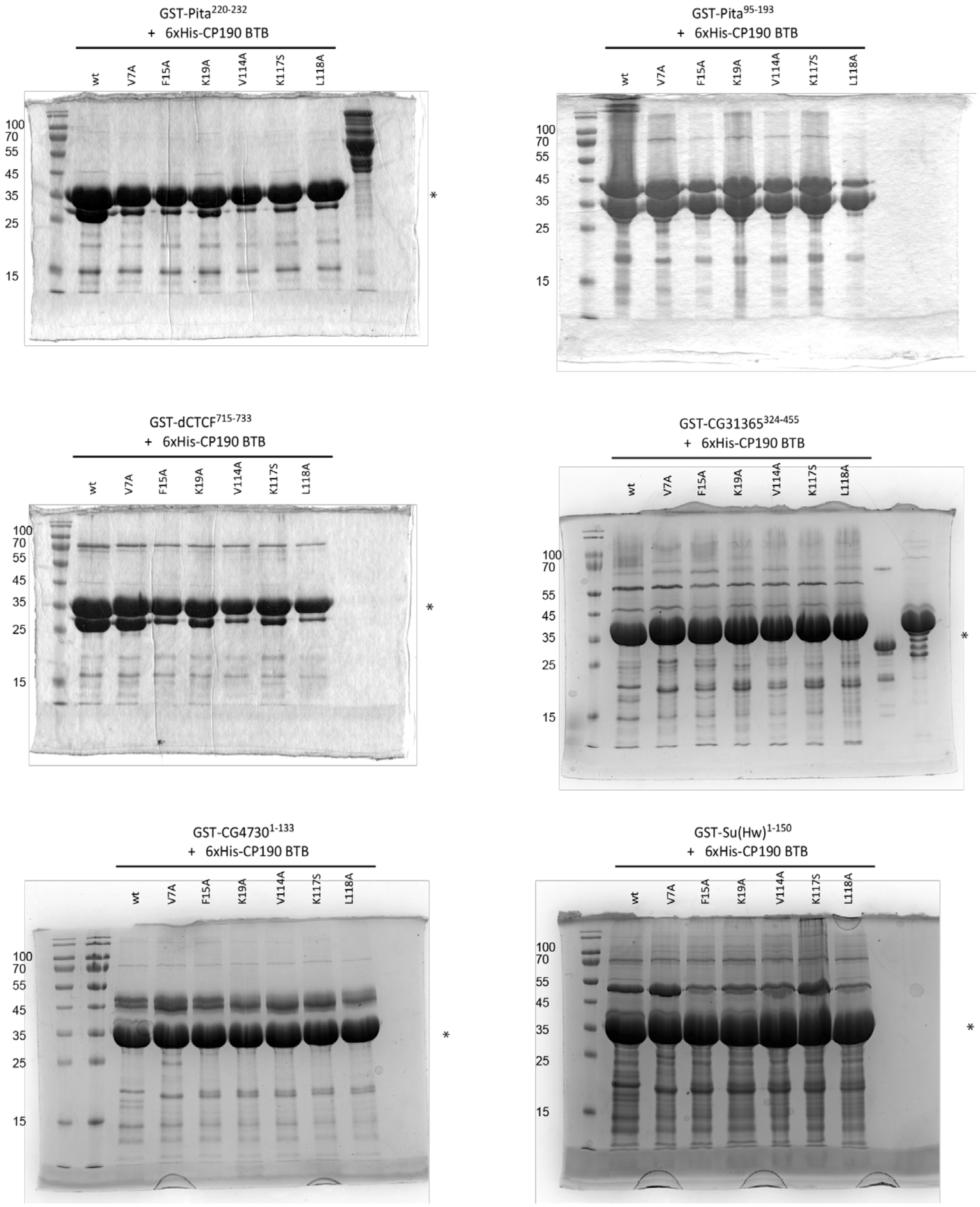

Figure S9. Testing of the impact of point mutations in CP190 BTB-domain on its interaction with architectural proteins using 6xHis-pulldown assay with bacterially expressed proteins. Position of CP190 BTB-domain is shown with asterisk.

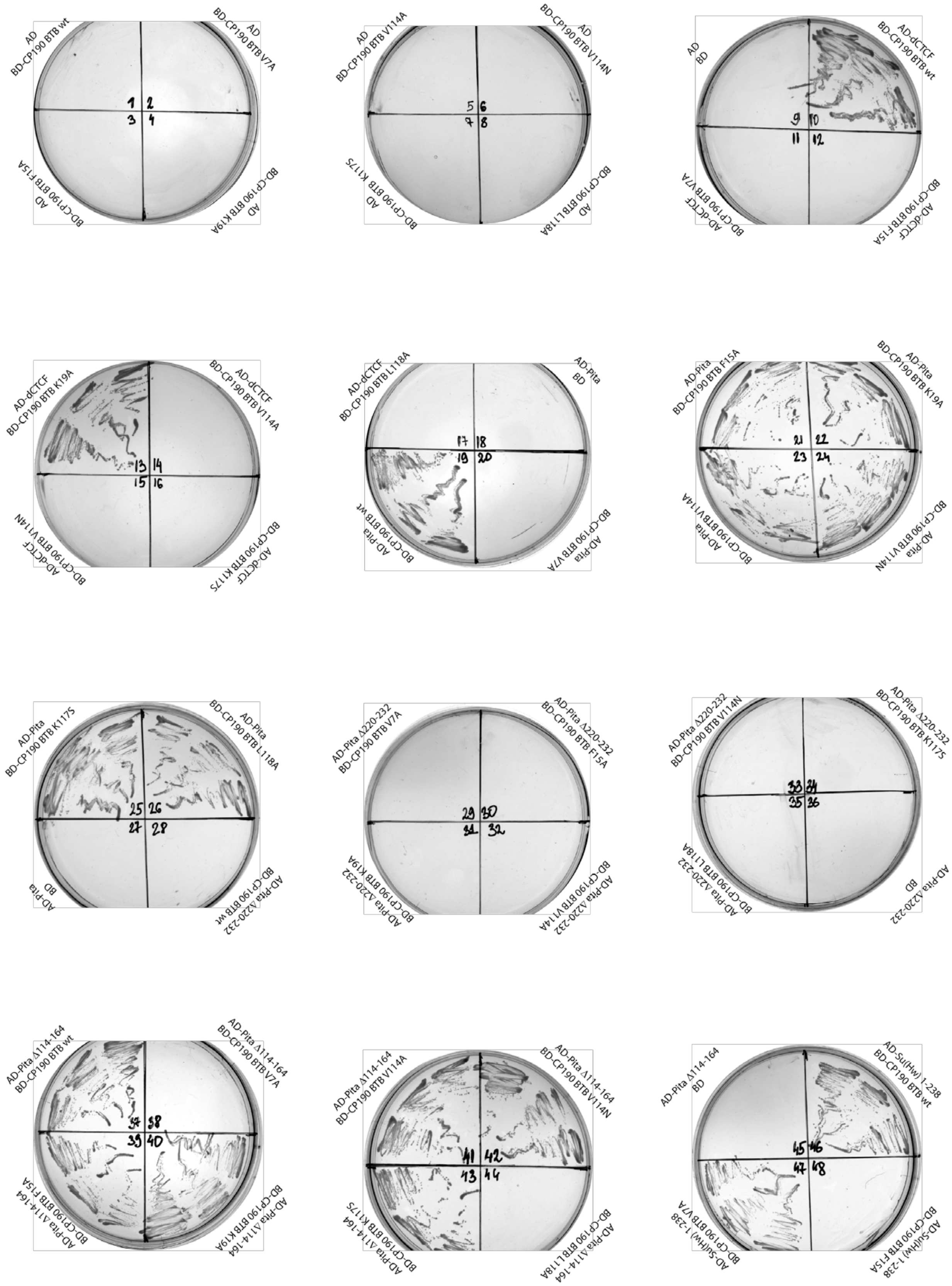
AD
BD-CP190 BTB wt
BD-CP190 BTB V7A
AD
BD-CP190 BTB F15A
AD
AD
BD-CP190 BTB K19A
AD
BD-CP190 BTB V114A
BD-CP190 BTB V114N
AD
BD-CP190 BTB K117S
AD
AD
BD-CP190 BTB L118A
AD
BD
AD-dCTCF
BD-CP190 BTB wt
AD-dCTCF
BD-CP190 BTB V7A
AD-dCTCF
BD-CP190 BTB F15A
AD-dCTCF
BD-CP190 BTB K19A
AD-dCTCF
BD-CP190 BTB V114A
AD-dCTCF
BD-CP190 BTB V114N
AD-dCTCF
BD-CP190 BTB K117S
AD-dCTCF
BD-CP190 BTB L118A
AD-Pita
BD
AD-Pita
BD-CP190 BTB wt
AD-Pita
BD-CP190 BTB V7A
AD-Pita
BD-CP190 BTB F15A
AD-Pita
BD-CP190 BTB K19A
AD-Pita
BD-CP190 BTB V114A
AD-Pita
BD-CP190 BTB V114N
AD-Pita
BD-CP190 BTB K117S
AD-Pita
BD-CP190 BTB L118A
AD-Pita
BD
AD-Pita Δ220-232
BD-CP190 BTB wt
AD-Pita Δ220-232
BD-CP190 BTB V7A
AD-Pita Δ220-232
BD-CP190 BTB F15A
AD-Pita Δ220-232
BD-CP190 BTB K19A
AD-Pita Δ220-232
BD-CP190 BTB V114A
AD-Pita Δ220-232
BD-CP190 BTB V114N
AD-Pita Δ220-232
BD-CP190 BTB K117S
AD-Pita Δ220-232
BD-CP190 BTB L118A
AD-Pita Δ220-232
BD
AD-Pita Δ114-164
BD-CP190 BTB wt
AD-Pita Δ114-164
BD-CP190 BTB V7A
AD-Pita Δ114-164
BD-CP190 BTB F15A
AD-Pita Δ114-164
BD-CP190 BTB K19A
AD-Pita Δ114-164
BD-CP190 BTB V114A
AD-Pita Δ114-164
BD-CP190 BTB V114N
AD-Pita Δ114-164
BD-CP190 BTB K117S
AD-Pita Δ114-164
BD-CP190 BTB L118A
AD-Pita Δ114-164
BD
AD-Su(Hw) 1-238
BD-CP190 BTB wt
AD-Su(Hw) 1-238
BD-CP190 BTB V7A
AD-Su(Hw) 1-238
BD-CP190 BTB F15A

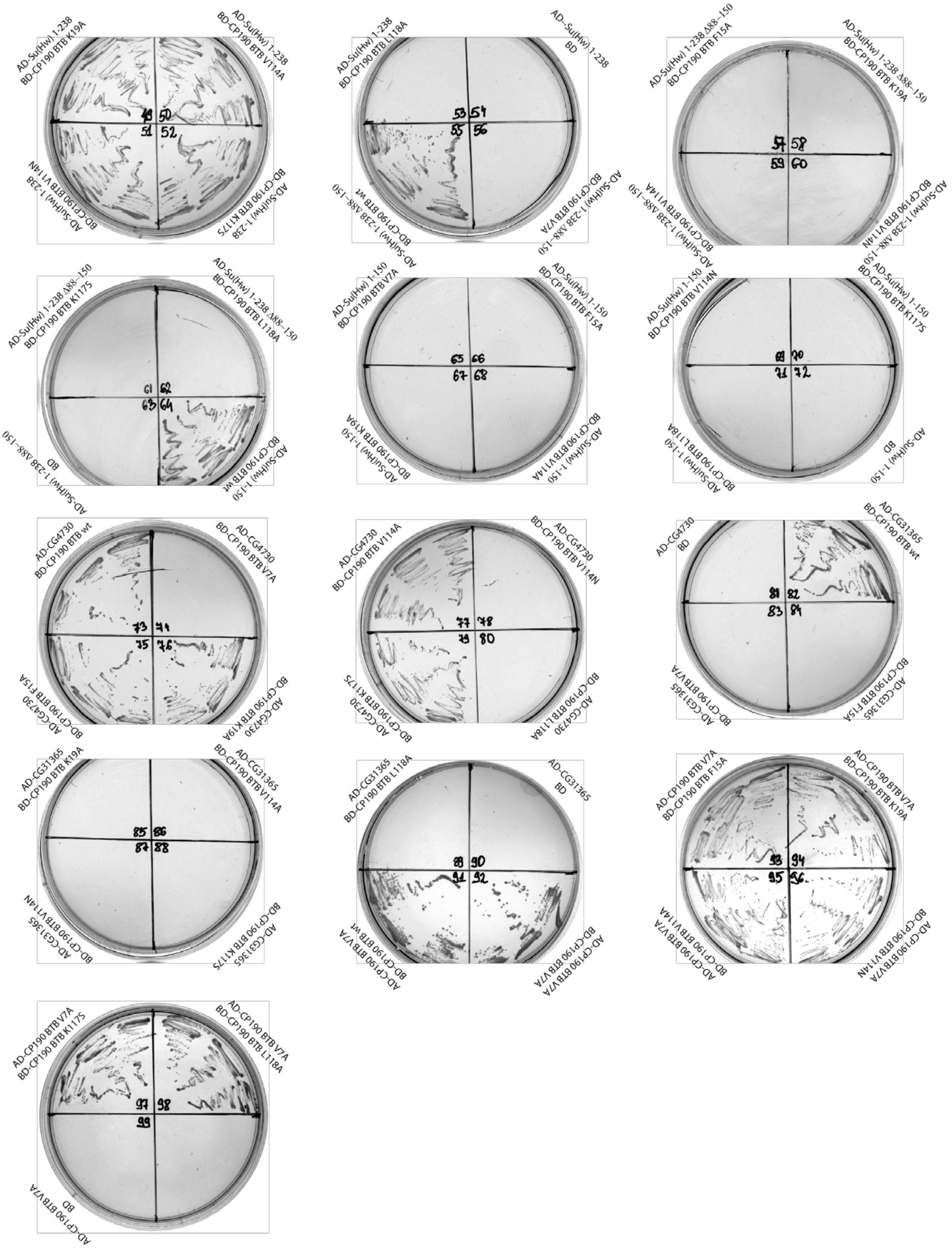

Figure S10. Testing the interactions between C2H2 proteins and wild-type or mutant CP190 BTB domains using yeast two-hybrid assay. Growth assay plates without histidine are shown (yeasts are unable to grow on this medium in the absence of interaction). AD stands for Activation Domain, BD – for DNA-Binding Domain of GAL4 protein.

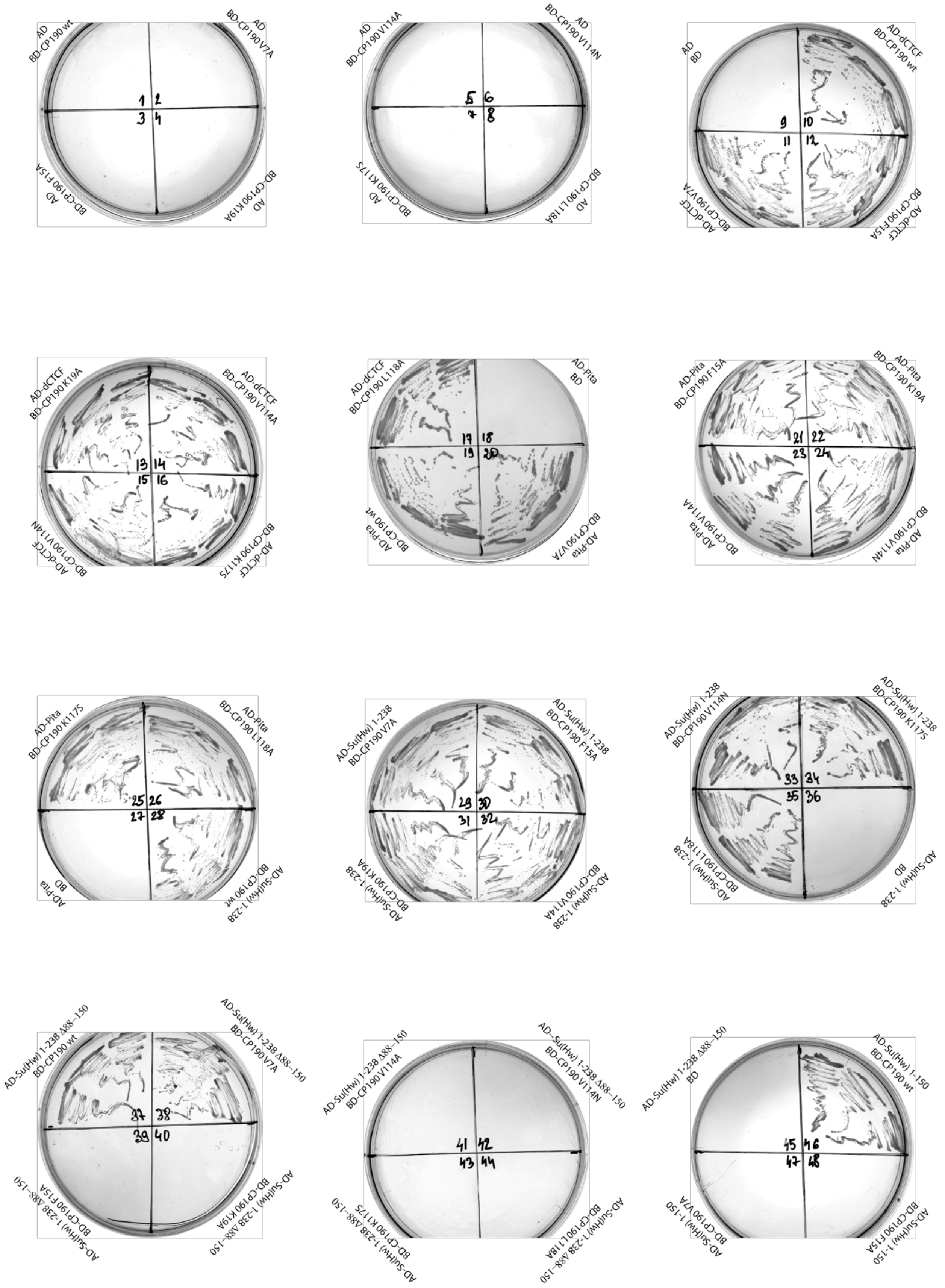

AD BD-CP190 wt
AD BD-CP190 V7A
AD BD-CP190 F15A
AD BD-CP190 K19A
AD BD-CP190 V114A
AD BD-CP190 V114N
AD BD-CP190 K117S
AD BD-CP190 L118A
AD BD
AD-dCTCF BD-CP190 wt
AD-dCTCF BD-CP190 V7A
AD-dCTCF BD-CP190 F15A
AD-dCTCF BD-CP190 K19A
AD-dCTCF BD-CP190 V114A
AD-dCTCF BD-CP190 V114N
AD-dCTCF BD-CP190 K117S
AD-dCTCF BD-CP190 L118A
AD-Pita BD
AD-Pita BD-CP190 wt
AD-Pita BD-CP190 V7A
AD-Pita BD-CP190 F15A
AD-Pita BD-CP190 K19A
AD-Pita BD-CP190 V114A
AD-Pita BD-CP190 V114N
AD-Pita BD-CP190 K117S
AD-Pita BD-CP190 L118A
AD-Pita BD
AD-Su(Hw) 1-238 BD-CP190 wt
AD-Su(Hw) 1-238 BD-CP190 V7A
AD-Su(Hw) 1-238 BD-CP190 F15A
AD-Su(Hw) 1-238 BD-CP190 K19A
AD-Su(Hw) 1-238 BD-CP190 V114A
AD-Su(Hw) 1-238 BD-CP190 V114N
AD-Su(Hw) 1-238 BD-CP190 K117S
AD-Su(Hw) 1-238 BD-CP190 L118A
AD-Su(Hw) 1-238 BD
AD-Su(Hw) 1-238 Δ88–150 BD-CP190 wt
AD-Su(Hw) 1-238 Δ88–150 BD-CP190 V7A
AD-Su(Hw) 1-238 Δ88–150 BD-CP190 F15A
AD-Su(Hw) 1-238 Δ88–150 BD-CP190 K19A
AD-Su(Hw) 1-238 Δ88–150 BD-CP190 V114A
AD-Su(Hw) 1-238 Δ88–150 BD-CP190 V114N
AD-Su(Hw) 1-238 Δ88–150 BD-CP190 K117S
AD-Su(Hw) 1-238 Δ88–150 BD-CP190 L118A
AD-Su(Hw) 1-238 Δ88–150 BD
AD-Su(Hw) 1-150 BD-CP190 wt
AD-Su(Hw) 1-150 BD-CP190 V7A
AD-Su(Hw) 1-150 BD-CP190 F15A

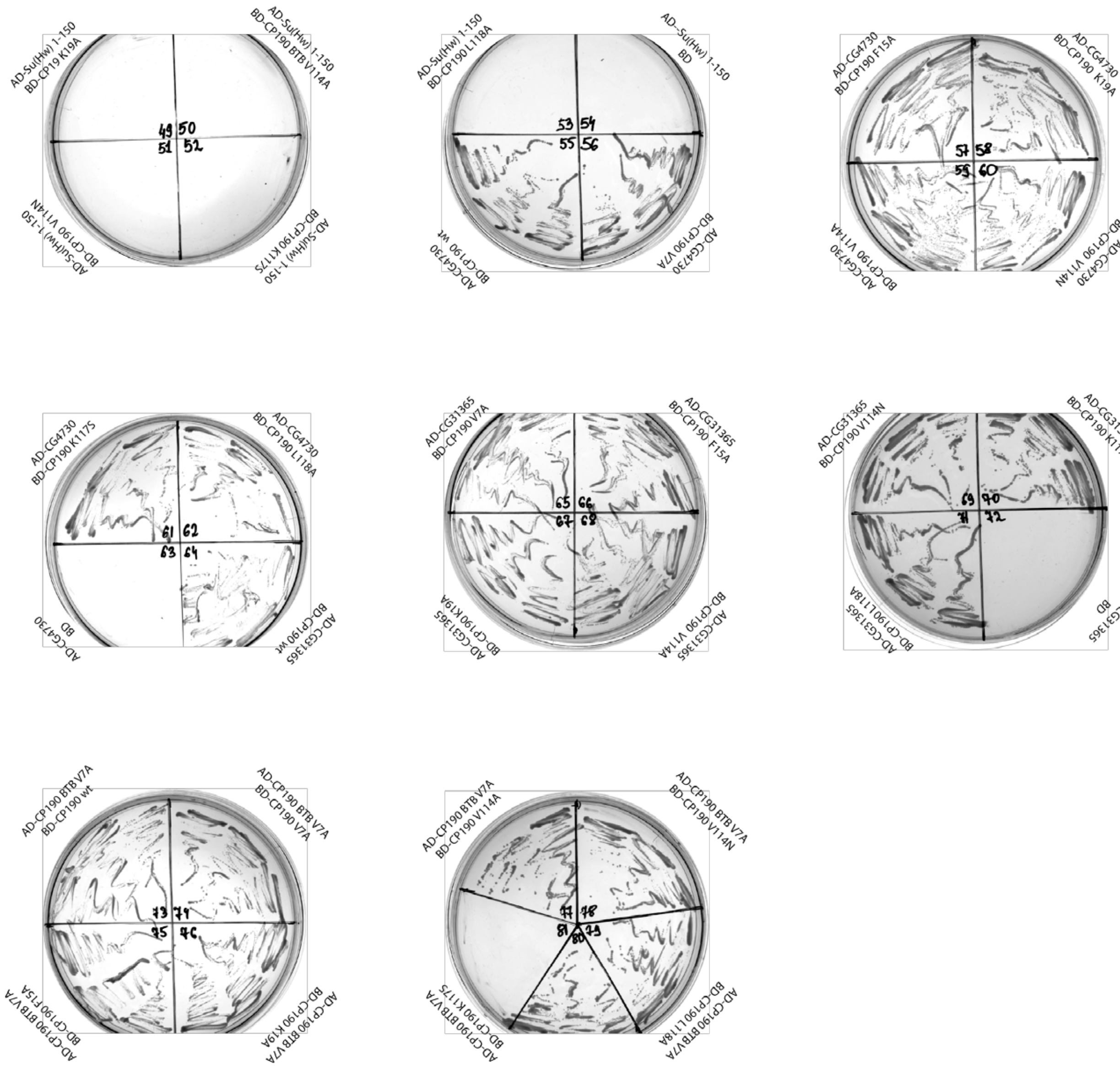

Figure S11. Testing the interactions between C2H2 proteins and full-length CP190 proteins bearing mutations within BTB domain using yeast two-hybrid assay. Growth assay plates without histidine are shown (yeasts are unable to grow on this medium in the absence of interaction). AD stands for Activation Domain, BD – for DNA-Binding Domain of GAL4 protein.

Supplementary references

1. Vogelmann, J.; Le Gall, A.; Dejardin, S.; Allemand, F.; Gamot, A.; Labesse, G.; Cuvier, O.; Negre, N.; Cohen-Gonsaud, M.; Margeat, E.; et al. Chromatin insulator factors involved in long-range DNA interactions and their role in the folding of the Drosophila genome. *PLoS Genet* **2014**, *10*, e1004544, doi:10.1371/journal.pgen.1004544.
2. Plevock, K.M.; Galletta, B.J.; Slep, K.C.; Rusan, N.M. Newly Characterized Region of CP190 Associates with Microtubules and Mediates Proper Spindle Morphology in Drosophila Stem Cells. *PLoS One* **2015**, *10*, e0144174, doi:10.1371/journal.pone.0144174.
3. Drosophila 12 Genomes, C.; Clark, A.G.; Eisen, M.B.; Smith, D.R.; Bergman, C.M.; Oliver, B.; Markow, T.A.; Kaufman, T.C.; Kellis, M.; Gelbart, W.; et al. Evolution of genes and genomes on the Drosophila phylogeny. *Nature* **2007**, *450*, 203-218, doi:10.1038/nature06341.
